# Using structured libraries, selection, and machine learning to rapidly explore the sequence space of a fluorescent deoxyribozyme

**DOI:** 10.1093/nar/gkaf1348

**Published:** 2025-12-12

**Authors:** Jaroslav Kurfürst, Martin Volek, Raman Samusevich, Tomáš Pluskal, Edward A Curtis

**Affiliations:** Institute of Organic Chemistry and Biochemistry of the Czech Academy of Sciences, Prague 166 10, Czech Republic; Department of Informatics and Chemistry, University of Chemistry and Technology, Prague 166 28, Czech Republic; Institute of Organic Chemistry and Biochemistry of the Czech Academy of Sciences, Prague 166 10, Czech Republic; Department of Genetics and Microbiology, Faculty of Science, Charles University in Prague, Prague 128 44, Czech Republic; Institute of Organic Chemistry and Biochemistry of the Czech Academy of Sciences, Prague 166 10, Czech Republic; Czech Institute of Informatics, Robotics and Cybernetics, Czech Technical University, Prague 160 00, Czech Republic; Institute of Organic Chemistry and Biochemistry of the Czech Academy of Sciences, Prague 166 10, Czech Republic; Institute of Organic Chemistry and Biochemistry of the Czech Academy of Sciences, Prague 166 10, Czech Republic

## Abstract

Finding ways to more comprehensively explore the sequence space of complex functional motifs is an important and unresolved question in nucleic acid engineering. Standard approaches use libraries in which a single variant of a motif is randomly mutagenized at a low level. This provides comprehensive coverage of sequence space over short mutational distances, but only limited information about more distant variants. Here we describe a new approach that uses libraries made up of sequences consistent with the multiple constraints of a desired target motif. Functional variants are rapidly identified in a single round of selection followed by high-throughput sequencing, and rules relating sequence to function are elucidated using machine learning. This method was tested using a fluorescent deoxyribozyme recently discovered in our group called Aurora. Single-step selections showed that a secondary structure library based on Aurora contained ~40-fold more unique catalytic sequences than one generated by random mutagenesis. Furthermore, models developed by machine learning could quantitatively predict read numbers and identify the most active variants using small subsets of sequences as training sets. By combining secondary structure libraries, selection, and machine learning in this way, sequence space can be explored far more quickly and efficiently than in standard approaches.

## Graphical Abstract

**Graphical Abstract:**
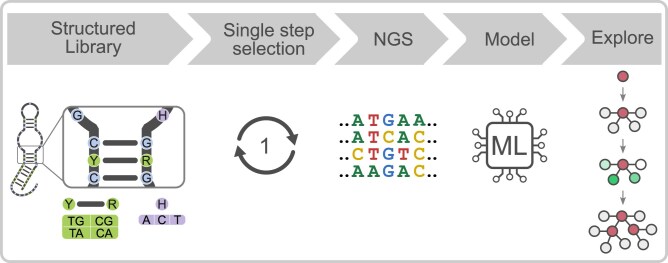

## Introduction

Methods such as SELEX and *in vitro* selection make it possible to isolate nucleic acid sequences with specific phenotypes from large random sequence libraries [1–3]. Many selections have been performed over the last three decades and have established that this approach is remarkably general [4–7]. However, a major unresolved challenge is that only a tiny fraction of sequence space can be searched using existing methods. This limitation can be seen most clearly when considering random sequence libraries: because an oligonucleotide of length N can have 4^N^ different sequences, the number of possible variants of even a modestly sized oligonucleotide is far greater than 10^16^ (which is the approximate size limit for a library in a selection experiment). However, it also applies to reselection experiments in which a library is prepared by randomly mutagenizing a single variant of a motif at a low level [3, 8]. Such libraries can contain all possible variants of the starting sequence over short mutational distances [9] but typically provide little information about more distant variants. Efforts to explore nucleic acid sequence space using machine learning have highlighted the importance of broader sampling [10–12]. For example, although clusters of similar sequences with a given fold (such as those generated by random mutagenesis and selection) can provide limited information about the activities of new variants, the accuracy of predictions generally decreases as mutational distance from the cluster increases [11, 12]. These considerations highlight the critical need for methods that increase both coverage and diversity in selection experiments.

**Figure 1.**
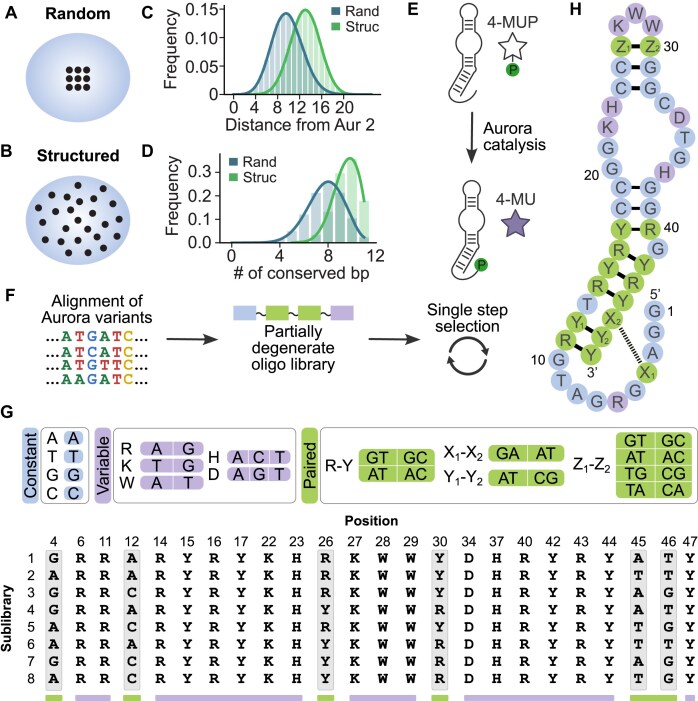
Exploring sequence space using secondary structure libraries. (**A**) Theoretical figure showing expected sampling in a randomly mutagenized library. The blue oval represents all possible sequences of a given length. Black circles represent sequences with the ability to form a particular secondary structure with some function. Distances between black circles are proportional to mutational distance, and the number of black circles is proportional to the number of unique sequences with the potential to form the desired secondary structure. (**B**) Same, but for a secondary structure library. Note that the blue ovals in panels (A) and (B) represent the same sequences, but the black circles in most cases do not. (**C**) Calculated distribution of mutational distances in a library generated by randomly mutagenizing Aurora 2 at a rate of 21% per position (blue) and in a secondary structure library (green) based on Aurora 2. (**D**) Same as panel (C), but showing the fraction of library members in which different numbers of base pairs in Aurora are conserved. (**E**) Reaction catalyzed by Aurora. (**F**) Workflow used to design the secondary structure library. (**G**) Strategies to encode highly conserved positions (blue), unpaired positions (purple), and positions that form base pairs (green) in the secondary structure of Aurora. The sequences of the variable positions in the eight oligonucleotides used to generate the Aurora secondary structure library are shown below. (**H**) Sequence of the library mapped onto the secondary structure of Aurora.

In this study we developed a new method to explore the sequence space of complex motifs and used it to rapidly characterize a recently discovered fluorescence-producing deoxyribozyme called Aurora [13]. One innovative aspect of this approach is the method of library construction. Rather than generating diversity by random mutagenesis, we instead utilized variation from previously characterized variants of a motif, and encoded base pairs using strategies that maximize diversity while minimizing the probability of disrupting the pair [14]. Such a strategy was expected to increase both the fraction and diversity of catalytically active sequences relative to those in a library generated by random mutagenesis (Fig. 1A–D). Consistent with these expectations, a secondary structure library constructed in this way contained ~40 times more active variants of Aurora than one generated by random mutagenesis. It could also be analyzed using single-step selections [15], which are quicker and easier than conventional ones that often require ten or more rounds. A second novel aspect of our approach is the use of machine learning to elucidate the complex patterns relating sequence to function. This enabled us to quantitatively predict CPM (counts per million) values and identify the most active variants using small subsets of sequences as training sets. Our results highlight the advantages of combining structured libraries and selection with machine learning. They also raise the intriguing possibility that this approach can be employed to comprehensively explore fitness landscapes that are too large to fully characterize by selection alone.

## Materials and methods

### Oligonucleotides

Oligonucleotides were chemically synthesized by GENERI BIOTECH s.r.o., Sigma–Aldrich, or IDT and purified by 7 M urea 6% PAGE or HPLC. See Supplementary Table S1 for the sequences of oligonucleotides used in this study.

### Library synthesis

Both the secondary structure library and the randomly mutagenized control library were encoded by eight sub-libraries, each encoded by a different partially degenerate oligonucleotide. In the case of the secondary structure library, these contained mutations consistent with the secondary structure and sequence requirements of Aurora, while in the case of the randomly mutagenized library, they contained randomly chosen mutations. Oligonucleotides were generated by solid-phase DNA synthesis using hand-mixed phosphoramidites and mixed in equimolar ratios to obtain the final libraries (see Supplementary Table S1, Secondary SubPools 1–8 and Random SubPools 1–8). Approximately 34% of the 2.8 × 10^7^ unique sequences encoded by the secondary structure library were detected by high-throughput sequencing (3.8 × 10^7^ unique reads), with read numbers ranging from 1 to 309, and CPM values ranging from 0.026 to 8.072. This range is significantly smaller when outliers are removed. For example, when the most abundant 1% of sequences were removed, read numbers ranged from 1 to 22, and when the most abundant 10% of sequences were removed, read numbers ranged from 1 to 8. Libraries were purified by 7 M urea 6% PAGE prior to use.

### Single-step selections

Each library was mixed with the blocking oligonucleotide REV1 in Milli-Q water. After heating at 65°C for 2 min and cooling at room temperature for 5 min, 5× Aurora buffer and 4-MUP were added. Final concentrations were 1 μM secondary structure library or control library (corresponding to 5.6 × 10^13^ molecules), 1.5 μM REV1, 1× Aurora buffer [200 mM KCl, 1 mM ZnCl_2_, 50 mM HEPES pH 7.4, and 5% (v/v) DMSO], and either 20 μM 4-MUP (*K*_M_ selection) or 100 μM 4-MUP (*k*_cat_ selection). This was incubated for 10 min in the *K*_M_ selection and 100 s in the *k*_cat_ selection. The incubation was stopped and the DNA was concentrated by ethanol precipitation. A short oligonucleotide (FWD1) was then ligated to reacted (5′ phosphorylated) library members. The efficiency of the ligation reaction was ensured by a splint oligonucleotide (Splint1), which was complementary to FWD1 and the 5′ end of both libraries. The ligation reaction was incubated for 5 min at 37°C. Final concentrations were 2.5 μM library, 2.5 μM FWD1, 2.5 μM Splint1, 1 × T4 DNA ligase buffer (Jena Bioscience), and 0.5 Weiss units of T4 DNA ligase (Jena Bioscience) per 1.0 μg of library. Reacted and unreacted DNA molecules were then separated by 7 M urea 6% PAGE. DNA molecules that co-migrated with an 87-long oligonucleotide marker were cut from the gel, eluted into 0.3 M NaCl, and ethanol precipitated. Purification on a 7 M urea 6% PAGE gel, elution, and ethanol precipitation were repeated to ensure better enrichment of active sequences. Library molecules were then amplified by polymerase chain reaction (PCR) using Q5 HotStart DNA Polymerase and the FWD1 and REV1 primers. Final concentrations were 500× diluted library, 0.5 μM FWD1, 0.5 μM REV1, 1× Q5 reaction buffer (NEB), 1× Q5 high GC enhancer (NEB), 0.2 mM dNTPs (Jena Bioscience), and 0.02 U of Q5 HotStart DNA polymerase (NEB) per 1 μl of the PCR reaction mixture. Double-stranded PCR products were isolated using a Macherey-Nagel PCR Clean-up Kit (Macherey-Nagel). Amplified libraries were sequenced by Eurofins Genomics using an amplicon paired-end sequencing run.

### Analysis of fluorescence production

To measure fluorescence production, oligonucleotides corresponding to individual sequences were resuspended in Milli-Q water, heated at 65°C for 2 min, and cooled at room temperature for 5 min. After adding 5× Aurora buffer, samples were transferred to a white half-area 96-well plate (Corning), and 4-MUP was then added. In a typical experiment, final concentrations were 15 μM of the tested oligonucleotide and 1× Aurora buffer [200 mM KCl, 1 mM ZnCl_2_, 50 mM HEPES pH 7.4, 5% (v/v) DMSO]. In the case of sequences from the *K*_M_ selection, the incubation time was 10 min and the 4-MUP concentration was 20 μM. In the case of sequences from the *k*_cat_ selection, the incubation time was 100 s and the concentration of 4-MUP was 100 μM. After incubating for the indicated time, reactions were quenched with 20 μl of 1 M KOH, and fluorescence was measured using a plate reader. Fluorescence was measured in white half-area 96-well plates (Corning) using a Tecan Spark plate reader with the following settings: excitation 358 (±5) nm, emission 455 (±5) nm, optimal gain, 30 flashes, and Z position calculated from one well in the plate.

### Analysis of phosphorylation

Analysis of self-phosphorylation was performed as described in Svehlova *et al.* 2022 [16]. Briefly, oligonucleotides corresponding to individual sequences were heated in Milli-Q water at 65°C for 2 min and cooled at room temperature for 5 min. They were then mixed with 5× Aurora buffer and 4-MUP. Final concentrations were 1 μM oligonucleotide, 1× Aurora buffer [200 mM KCl, 1 mM ZnCl_2_, 50 mM HEPES pH 7.4, and 5% (v/v) DMSO], and 1 mM 4-MUP or DiFMUP unless stated otherwise. After incubating for the indicated time, reactions were stopped by adding ethylenediaminetetraacetic acid (EDTA), and DNA was concentrated by ethanol precipitation. Reacted oligonucleotides (now containing a 5′ phosphate) were then ligated to a short oligonucleotide. Reacted and unreacted oligonucleotides were separated by 7 M urea 6% PAGE. DNA was visualized by staining with GelRed and gels were scanned using a Typhoon laser scanner. The amount of reacted and unreacted oligonucleotide in each reaction was quantified using ImageQuant TL software.

### Kinetics measurements and analysis

Kinetic measurements, data analysis, and curve fitting were performed as described in Svehlova *et al.* 2022 [16] and Jakubec *et al.* 2022 [17]. Briefly, variants of Aurora (specifically the Aurora 34T and Aurora 34G variants) were mixed with Milli-Q water, heated at 65°C for 2 min, and cooled at room temperature for 5 min. Aurora was then mixed with 5× Aurora buffer and 4-MUP. Final concentrations were 1 μM Aurora, 1× Aurora buffer [200 mM KCl, 1 mM ZnCl_2_, 50 mM HEPES pH 7.4, and 5% (v/v) DMSO], and 1 μM to 300 μM 4-MUP. Reactions were incubated at room temperature for the indicated time and stopped by adding EDTA to a final concentration of 25 mM. Reactions were stopped at time points that corresponded to the linear phase of the reaction. Aurora was purified by ethanol precipitation. Reacted (5′ phosphorylated) Aurora was then ligated to a short oligonucleotide as described in the section “Single-step selections.” Reacted and unreacted molecules were separated by 7 M urea 6% PAGE. Aurora was visualized by staining with GelRed. Gels were scanned using a Typhoon laser scanner. The amount of reacted and unreacted Aurora was quantified using ImageQuant TL software. See Supplementary Table S1 for the sequences of deoxyribozymes used in kinetic assays.

### Calculation of signal-to-noise ratios

Signal-to-noise ratios were defined as the fluorescence of a sample in the presence of deoxyribozyme divided by the fluorescence of the sample in the absence of deoxyribozyme. The background signal was defined as the fluorescence of 1× Aurora buffer [200 mM KCl, 50 mM HEPES, pH 7.4, 1 mM ZnCl_2_ and 5% (v/v) DMSO] and was subtracted before calculating signal-to-noise ratios.

### Next-generation sequencing and data analysis

All libraries were sequenced by Eurofins Genomics using amplicon paired-end sequencing runs. Raw reads were processed using a pipeline consisting of adaptor trimming (Cutadapt v1.18), read merging (fastq-join v1.3.1), unifying of read orientation (fastx barcode splitter), primer clipping (Cutadapt v1.18), length filtering (Cutadapt v1.18), and counting of unique sequences (bash). All further analysis was performed using in-house Python scripts available at https://doi.org/10.6084/m9.figshare.30490904.

### Developing predictive models using machine learning

Two different machine learning workflows were used to analyze selection data (Supplementary Fig. S1). In the first workflow, in which the fitness landscape was modeled, the dataset was split by randomly selecting a fraction of 0.1 of the sequences for training and a different, disjunct 0.1 fraction for testing. In the second workflow, data were split into training, validation, and testing sets based on fitness. The sequences with the 100 highest CPM values were used as the testing set, the sequences with the next 100 highest CPM values were used as the validation set, and the remaining sequences were used as the training set. Training data were also randomly sampled using 1.0, 0.1, 0.01, and 0.001 fractions. All models were fully connected feedforward neural networks. The neural networks were implemented in Python 3.10.0, using the scikit-learn library (v1.5.1). Two phases of optimization took place. First, a grid search was used for hyperparameter tuning of the neural network with a fixed set of parameters for the beam search: “topN_start”: 5, “beam_width”: 5, “max_depth”: 5, “top_explored”: 100, “mode”: “directed.” The metric optimized for was precision of the end-to-end pipeline and the grid consisted of the following values: “hidden_layer_sizes”: [(50, 50), (50, 50, 50), (100, 100), (100, 100, 100), (200, 200, 200), (100, 100, 100, 100)], “learning_rate_init”: [0.01, 0.001, 0.0001], “batch_size”: [100, 200]. The data used for this optimization was the validation set and the 0.01 fraction sampling of the training data. Once the neural network hyperparameters were optimized, different parameters of the beam search algorithm (topN_start, “beam_width,” “max_depth”) were explored as well. With optimized hyperparameters the final neural network was trained on combined training and validation sets and sequences generated using the optimized beam search were then evaluated against the holdout set. The source code to the pipeline and all the analysis can be found on Figshare at https://doi.org/10.6084/m9.figshare.30490904.

## Results

### A secondary structure library based on a fluorescent deoxyribozyme

Aurora is a deoxyribozyme recently discovered in our group [13]. It generates a fluorescent product by transferring the phosphate group from the coumarin substrate 4-MUP to its own 5′ hydroxyl group (Fig. 1E). Other deoxyribozymes generate chemiluminescent [16, 17] or colorimetric [18] signals using similar mechanisms. We previously performed a reselection experiment [8, 19, 20] that provided information about the secondary structure and sequence requirements of Aurora [13]. Here we used solid-phase DNA synthesis and several different coding strategies to construct a library enriched for sequences consistent with these constraints (Fig. 1F). Conserved nucleotides were not mutated during DNA synthesis and were encoded using invariant positions (Fig. 1G, blue). Unpaired variable positions were encoded using single degenerate positions (Fig. 1G, purple) [21], while constrained base pairs were generally encoded using pairs of degenerate positions such as R-Y (where R is A or G and Y is C or T) (Fig. 1G, green) [14]. Some patterns could not be encoded in a single synthesis. For instance, to encode an unconstrained base pair such that AT, TA, CG, GC, GT, or TG could occur, two different oligonucleotides were required: one that contained R-Y at these positions and one that contained Y-R at these positions (Fig. 1G, green) [14]. To account for all combinations of such patterns, eight different partially degenerate oligonucleotides were synthesized and mixed to produce the final library (Fig. 1G, below). This encoded 2.8 × 10^7^ unique sequences, 17.8% of which had the ability to form each of the 11 base pairs in Aurora (Fig. 1H), and contained all possible combinations of 35 mutations at 24 different positions (Fig. 1H). These corresponded to mutations frequently observed in sequences from a previous Aurora reselection experiment [13]. A randomly mutagenized control library was also constructed. This contained the same number of mutations and mutated positions as the secondary structure library, but the identities and positions of these mutations were randomly chosen (Supplementary Fig. S2). Of the 24 mutated positions in this library, 15 corresponded to positions that were also mutated in the secondary structure library. However, because the mutations encoded at these overlapping positions were in almost all cases different (compare Fig. 1H and Supplementary Fig. S2), the two libraries encoded only 128 common sequences. Both the secondary structure library and the randomly mutagenized control library also encoded Aurora 2 (the most active previously identified variant of Aurora). By monitoring the enrichment of this internal control, a selection that failed due to technical reasons could be distinguished from one that yielded few active variants due to the design of the library.

### The secondary structure library was enriched for functional sequences

**Figure 2.**
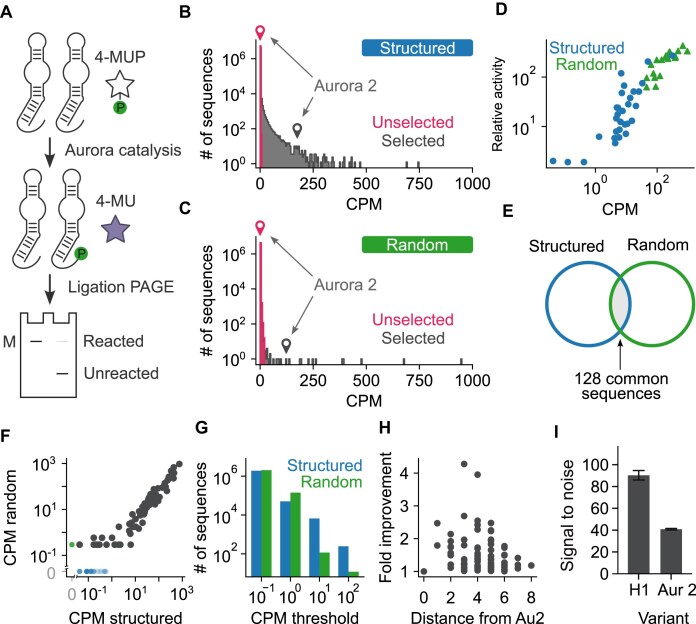
A secondary structure library based on Aurora is enriched for functional sequences. (**A**) Workflow of single-step selection experiments. After gel purification, sequences are excised from the gel, eluted, and ethanol precipitated, amplified by PCR, and characterized by high-throughput sequencing. (**B**) Distribution of CPM (counts per million) values in the secondary structure library before and after selection. The CPM value of Aurora 2 (the internal positive control and most active previously identified variant of Aurora) in the unselected library (red) and selected library (gray) is also shown. (**C**) Same as panel (B), but for the randomly mutagenized control library. (**D**) Correlation between CPM value and catalytic activity among sequences identified in selections for improved *K*_M_ values. Relative activity indicates the percent activity relative to Aurora 2. (**E**) Calibration of CPM values using the 128 sequences that are encoded by both the secondary structure library and the randomly mutagenized library. (**F**) Relationship between CPM values in the secondary structure library and CPM values in the randomly mutagenized library for the 128 sequences encoded by both libraries. Green and blue points correspond to sequences that were only observed in one of the two selected libraries, while black points correspond to sequences that were observed in both selected libraries and used for calibration. (**G**) Number of unique sequences in the secondary structure library (blue) and randomly mutagenized control library (green) with CPM values above different cutoffs. (**H**) Distribution of sequences in the secondary structure library with CPM value higher than that of Aurora 2 as a function of mutational distance from Aurora 2. (**I**) Catalytic rate of the most active variant of Aurora in the secondary structure library (H1) compared to Aurora 2 (Aur 2) under the conditions used in the selection.

Deoxyribozymes were isolated from both the secondary structure and control library by incubating with 4-MUP, tagging reacted (5′ phosphorylated) molecules by ligation, isolating tagged molecules using two consecutive PAGE purifications, and characterizing by high-throughput sequencing (Fig. 2A) [15]. Each sequence was ranked by its CPM value, which was defined as its read number (multiplied by one million) divided by the total number of reads. Similar results were obtained when sequences were ranked by enrichment values (defined as the frequency of a sequence in the evolved library divided by its calculated frequency in the starting library; Supplementary Fig. S3), suggesting that biases were small during library synthesis. Active sequences were significantly more abundant in the secondary structure library than in the randomly mutagenized one, while the Aurora 2 internal control was highly enriched in both selections (Fig. 2B and C). CPM values were correlated with catalytic activity over a wide range (Fig. 2D). This is likely because only one round of selection was required, which minimized biases from steps such as PCR. CPM values of the 128 sequences encoded by both libraries were strongly correlated in the two selections over the range of ∼1 CPM to 1000 CPM (Fig. 2E and F) indicating that these values could be quantitatively compared. Such a comparison revealed that catalytically active sequences were more than an order of magnitude more abundant in the secondary structure library than in the randomly mutagenized one (Fig. 2G and Supplementary Table S2). For example, 6585 sequences in the secondary structure library had a CPM value of 10 or more, whereas only 117 did in the randomly mutagenized library. This difference was even more pronounced when the 128 sequences common to both datasets were removed from the analysis (Supplementary Fig. S4 and Supplementary Table S2), indicating that many of the active variants in the control library correspond to sequences that by chance are consistent with the sequence requirements of Aurora rather than new variants of Aurora or distinct catalytic motifs. Sequences with CPM values higher than that of Aurora 2 occurred in both libraries, but were more abundant in the secondary structure library (compare Fig. 2B with 2C, and also see Fig. 2H). Biochemical assays provided additional support for these results and showed that the rates of variants with the highest CPM values were several times higher than that of Aurora 2 under the conditions used in the selection (Fig. 2I). Taken together, these results demonstrate that secondary structure libraries can be highly enriched for active and improved deoxyribozyme variants relative to those generated by random mutagenesis.

### Rapid identification of a mutation that changes substrate affinity and specificity

**Figure 3.**
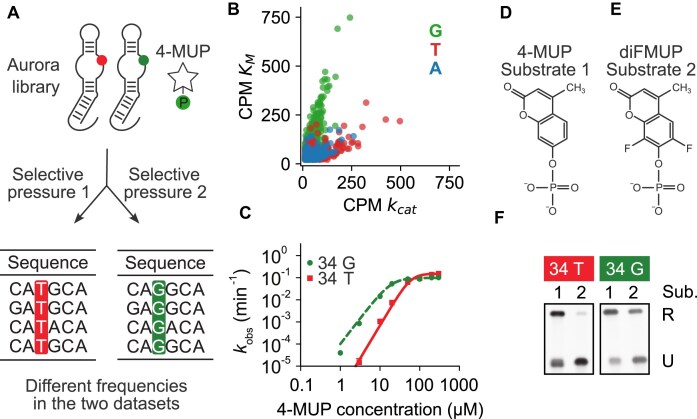
Rapid identification of a mutation that changes Aurora substrate affinity and specificity. (**A**) Experimental workflow. The 34G mutation is represented by a green circle, and the 34T mutation is represented by a red circle. (**B**) CPM values of sequences after a single-step selection for variants of Aurora with improved *k*_cat_ values (library incubated for 100 s in the presence of 100 µM 4-MUP; *x*-axis) and after a single-step selection for variants of Aurora with improved *K*_M_ values (library incubated for 10 min in the presence of 20 µM 4-MUP; *y*-axis). Note that the slope of the line formed by variants containing 34G (green points) is different from the slope of the line formed by variants containing 34T (red points). (**C**) Catalytic activity of Aurora variants containing 34G (green circles) or 34T (red squares) over a range of 4-MUP concentrations. (**D**) Chemical structure of 4-MUP. (**E**) Chemical structure of diFMUP. (**F**) Catalytic activity of the 34G (green) and 34T (red) variants of Aurora in the presence of 4-MUP and diFMUP. Reactions in panel (F) were incubated for 60 min in the presence of 1 mM of either 4-MUP or diFMUP and analyzed using a ligation assay.

After establishing that our approach can be used to rapidly generate large datasets of functional sequences, we next asked if it could also provide information about mutations that affect specific biochemical properties of a motif (Fig. 3A). We specifically focused on mutations that affect *k*_cat_ and *K*_M_ differently (see also reference [22]). In our initial selection (described earlier), the secondary structure library was incubated for 10 min at a subsaturating substrate concentration of 20 μM to favor variants with improved *K*_M_ values. To obtain additional information, we incubated the library for a shorter time (100 s) at a saturating substrate concentration (100 μM) to favor variants with improved *k*_cat_ values. CPM values of individual sequences were in most cases similar in these two selections (Fig. 3B), probably because mutations that affect general properties like stability affect *k*_cat_ and *K*_M_ values in similar ways. However, further analysis revealed two populations of sequences in which the slopes of the lines relating CPM value in the *k*_cat_ selection to that in the *K*_M_ selection were different (Fig. 3B). These populations corresponded to sequences with different nucleotides at position 34 in the asymmetric bulge of the deoxyribozyme (Fig. 3A). Variants with high CPM values in the *k*_cat_ selection but not the *K*_M_ selection usually contained T at this position (red points in Fig. 3B), while variants with high CPM values in both selections often contained G instead (green points in Fig. 3B). Catalytic assays confirmed the importance of position 34: a variant of Aurora containing T at position 34 was slightly more active at saturating concentrations of 4-MUP, whereas one containing G was ∼100-fold more active at subsaturating concentrations (Fig. 3C). This 100-fold difference in *k*_obs_ corresponded to a four-fold increase in the signal-to-noise ratio of fluorescence at subsaturating 4-MUP concentrations. Mutations at position 34 also affected the ability of Aurora to use the substrate diFMUP, an analog of 4-MUP that contains two fluorine atoms flanking the phosphate group, which are not present in 4-MUP (Fig. 3D–F). These observations suggest that position 34 forms part of the substrate binding pocket of Aurora. They also show how single-step selections performed under different conditions can provide rapid information about mutations that affect specific biochemical properties of a functional motif.

### The fitness landscape of Aurora contains both smooth and rugged features

**Figure 4.**
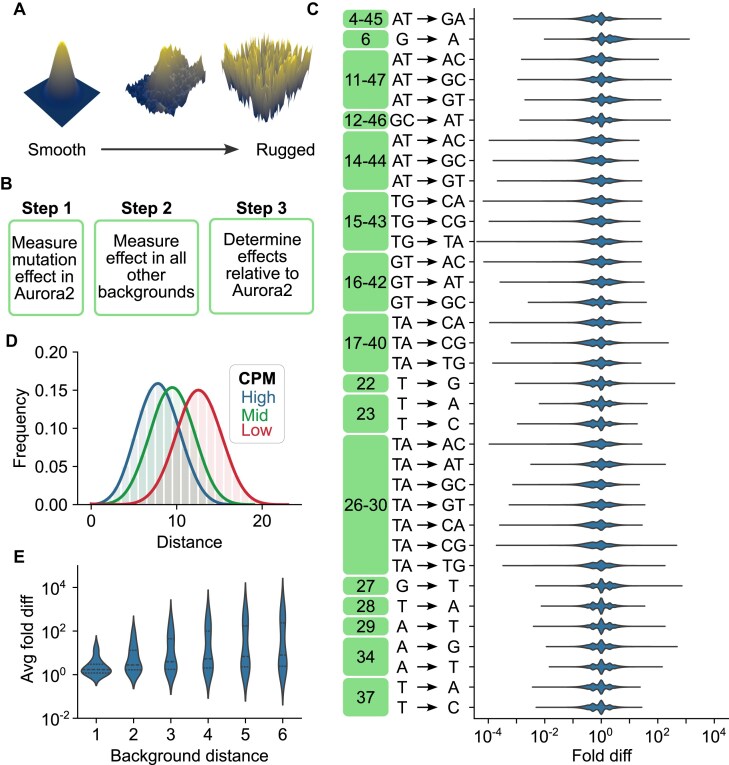
Mapping epistasis in Aurora. (**A**) Theoretical relationship between the degree of epistasis and the ruggedness of a fitness landscape. (**B**) Workflow to measure epistasis using Aurora 2 as a reference. (**C**) Effects of mutations relative to their effect in Aurora 2 vary significantly in different backgrounds. (**D**) Distribution of pairwise distances among sequences with high CPM values (blue), medium CPM values (green), and low CPM values (red). (**E**) Average effect of each point mutation encoded by the library compared to the effect in Aurora 2 as a function of the mutational distance of the sequence background from that of Aurora 2. Base pairs and the 4–45 noncanonical pair were treated as single positions in this analysis.

A fitness landscape describes the relationship between genotype and phenotype for all possible variants of a functional motif. Knowledge of the general properties of fitness landscapes can facilitate biopolymer engineering and optimization and has important implications for biological evolution [23]. The topology of a fitness landscape is strongly influenced by epistasis (Fig. 4A), which at its simplest level can be assessed by measuring the effect of a mutation over a range of sequence backgrounds (Fig. 4B). In the case of Aurora, epistasis was observed at every variable position in the library, and mutational effects generally varied by three to four orders of magnitude in different backgrounds (Fig. 4C). Analysis of pairwise correlations in Aurora by mutual information [19, 24] provided additional evidence for epistasis (Supplementary Fig. S5). Although these results indicate that the fitness landscape of Aurora is rugged (see also refs [25–28]), it also contains smooth features. Of particular interest is the observation that catalytically active sequences are clustered rather than randomly distributed (Fig. 4D). This suggested to us that information from active sequences could be used to predict the activities of similar sequences, and further analysis confirmed this possibility. In backgrounds that differ from Aurora 2 by a single mutation, for instance, average mutational effects of ∼80% of the mutations encoded by the library differ by less than five-fold from their effects in Aurora 2 (Fig. 4E and Supplementary Fig. S6). Similar results were observed for other sequence backgrounds (Supplementary Fig. S7). Taken together, these results indicate that, while the sequence landscape of Aurora is rugged, it also contains smooth features that enable prediction of mutational effects.

### Improved predictions using machine learning

**Figure 5.**
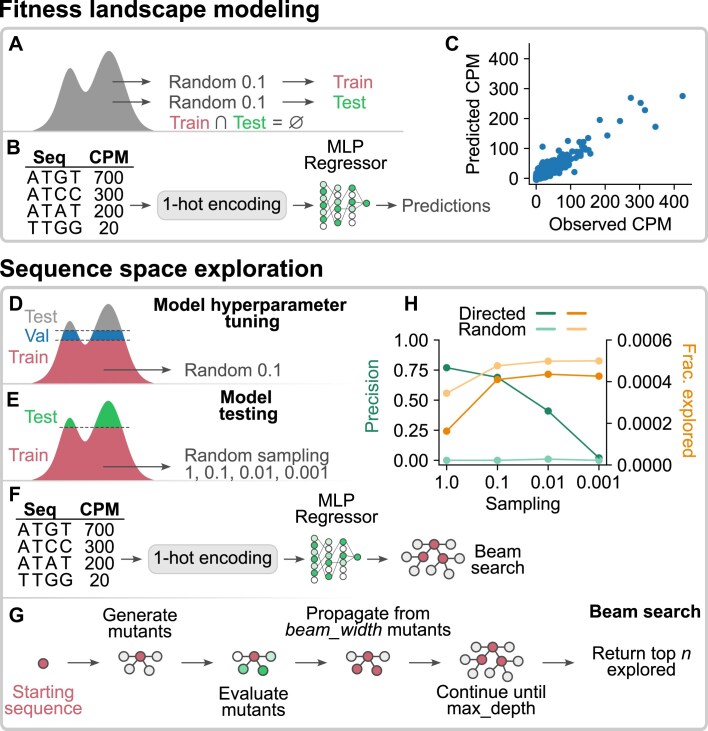
Prediction of mutational effects using machine learning. (**A**) Generation of a training set and a testing set (each containing a fraction of 0.1 of the dataset) by random sampling. These were chosen in such a way that they did not contain any common sequences. (**B**) The input training sequences were first 1-hot encoded and then used along with their CPM labels to train an MLPRegressor. Predictions were made on the 1-hot encoded testing sequences. (**C**) Correlation between predicted and experimentally observed CPM values. (**D**) To build and optimize the hyperparameters of the model, which was later used to guide the beam search, the dataset was split into a testing set (consisting of the top 100 sequences), a validation set (consisting of the next 100 sequences), and training sets with varying degrees of sampling (fractions of 1, 0.1, 0.01, or 0.001 of the remaining dataset). (**E**) Once models were optimized and a single model chosen, validation data were moved into the training set, and the model was trained again. (**F**) Unlike in panel (B), this final model was then used to guide a beam search algorithm. (**G**) Details of the beam search algorithm. (**H**) Precision and efficiency of the model-guided beam search compared to that of a control beam search in which decisions about direction of search propagation were made randomly. Precision is defined as the fraction of the 100 sequences in the testing set that were among the top 100 sequences found on the search tree. Fraction explored indicates the size of the search tree divided by the number of sequences that are encoded but not represented in the dataset.

The ability to predict mutational effects to a limited extent using simple distance-based rules raised the possibility that more complex models could achieve quantitative predictions. Inspired by recent developments in fields such as protein structural prediction [29], we hypothesized that such models could be developed using machine learning [30, 31]. This was further explored using a simple workflow (Fig. 5A and Supplementary Fig. S1). In the first step, a fraction of 0.1 of the sequences in the *K*_M_ dataset (corresponding to 750 379 unique sequences) was randomly chosen as a training set and a fraction of 0.1 as a testing set. Training and testing sets did not contain any common sequences. A fully connected feed-forward neural network was trained on the training set and then used to predict CPM values of sequences in the testing set (Fig. 5B). Predictions correlated well with experimental results (Fig. 5C), indicating that a model of this type can identify patterns needed for prediction. Smaller training sets generally yielded less accurate predictions (Supplementary Fig. S8 and Supplementary Table S3). We next attempted a more difficult task. The idea was to simulate a case in which only a subset of sequences encoded by a library are actually present during a selection and the missing subset contains some of the best sequences. (Fig. 5D–G and Supplementary Fig. S1). This could be the case when optimizing a complex motif in a conventional selection or a simpler motif in a single-step selection. The goal was to train a model on a subset of sequences represented in the dataset and attempt to find sequences that are better but missing from the dataset. We first removed the sequences with 100 highest CPM values from the dataset to be used as the testing set and the sequences with the next 100 highest CPM values to be used as the validation set. Subsets of various sizes of the remaining sequences were used as training data. We then implemented a beam search algorithm, which explored sequence space with guidance from the trained models. This started with the best sequences in the training set, generated neighboring sequences with single mutation steps, evaluated these mutants with the trained model, and selected several of the best mutants (the number of which was determined by the value of the beam_width parameter) to further propagate the search (Fig. 5G). This was repeated until a defined maximum search depth was achieved (determined by the value of the max_depth parameter). The result of a single run of this algorithm is a list of the top 100 sequences (in terms of predicted CPM) that were a part of the search tree. To evaluate the efficacy of the workflow, a precision metric was computed, which indicated the fraction of the top 100 sequences that were found in the testing or validation datasets. Note that in this case, precision and recall have the same value by definition. The neural network hyperparameters were optimized by testing different outcomes (runs of the beam search algorithm with models trained with various hyperparameter combinations) against the validation set. Once we found a good hyperparameter combination, we retrained the models on a merge of the training and validation sets and tested this against the top 100 sequences (which, as mentioned earlier, were initially removed from the dataset to be used as the testing set). Precision values of ∼0.4 could be achieved when only 1% of the dataset (corresponding to 75 036 unique sequences) was used for training (Fig. 5H and Supplementary Table S4) and were strongly dependent on the value of the max_depth parameter (Supplementary Fig. S9). Because this workflow minimizes the number of sequences that need to be evaluated relative to a naive search, it should be especially useful in cases in which large numbers of mutants need to be computationally analyzed. These two distinct workflows demonstrate that machine learning can be used to develop models that can quantitatively predict CPM values of variants in datasets generated from secondary structure libraries. They also raise the intriguing possibility that this approach can be used to fill in the gaps of fitness landscapes that are too large to analyze using experimental methods alone.

## Discussion

Sequence space is vast, and the initial isolate of a functional motif isolated from a random sequence library typically represents one of many sequences consistent with the motif. By randomly mutagenizing such a sequence at a low level and performing another selection, it is generally possible to identify clusters of similar sequences with the same secondary structure and function [3, 8, 13, 16, 18]. However, this approach is not an efficient way to identify variants of a motif that differ significantly from the original isolate [9, 14]. With this limitation in mind, we recently developed a method of library construction that makes it possible to more systematically explore the sequence space of a functional motif [14]. This uses libraries in which mutations are not random, but instead are consistent with the known secondary structure and sequence constraints of a motif of interest. Previous analysis suggested that libraries constructed in this way can contain orders of magnitude more sequences with the potential to form a desired motif than randomly mutagenized ones [14]. Here we provide experimental support for this idea: the frequency of catalytically active sequences in a secondary structure library based on the fluorescence-producing deoxyribozyme Aurora was ~40-fold higher than in one generated by random mutagenesis. These observations highlight the advantages of using secondary structure libraries to explore sequence space.

A second important point relates to the time and effort needed to identify functional variants of a motif. Because the libraries used in this study contained only ∼10^7^ unique sequences, selections could be performed in a single round followed by high-throughput sequencing [15]. This is a significant advantage relative to conventional selections, which can require ten or more rounds. While such single-step selections of the sort described here cannot be used to discover motifs that are too complex to occur in libraries of ∼10^7^ sequences, we suggest that they can greatly facilitate the rapid characterization of existing motifs, especially when combined with secondary structure libraries [14]. As a proof-of-concept, we used this approach to rapidly identify mutations that affect the substrate specificity of Aurora. It is likely that this approach could be used to facilitate identification of Aurora variants with other interesting properties and also be applied to the characterization of motifs other than Aurora.

A final point relates to the goal of finding the global optimum of a functional motif [23]. This is a significant challenge because the number of possible sequences with the potential to form a particular secondary structure is often considerably larger than the number of sequences that can be generated in a library. For this reason, developing methods to predict the activities of all possible sequence variants of a motif using information from a subset of them would be of great utility [10–12]. Here we have taken an important step toward this goal by showing that, despite the extremely high levels of epistasis in this landscape, the effects of mutations in Aurora can often be predicted using information about their effects in similar sequence backgrounds. Moreover, quantitative predictions can be achieved when datasets from secondary structure libraries are analyzed using machine learning. In the case of Aurora, randomly chosen fractions of 0.1 of the sequences in the dataset were sufficient to predict the activities of sequences not present in the training set, and even smaller subsets of sequences were sufficient to identify many of the most active variants. An important question is the extent to which our approach is general. A recent study from our group showed that, as was the case for Aurora, a secondary structure library based on the chemiluminescent deoxyribozyme Supernova [16] contained more than an order of magnitude more catalytic sequences than a randomly mutagenized one (Svehlova *et al.*, submitted). It was also possible to develop a model that could predict CPM values of sequences after selection. To our knowledge, these are the first two examples of such an approach being applied to deoxyribozymes. Additional examples will be needed to further establish the generality of this approach. If it is general, our approach has the potential to greatly facilitate optimization of catalytic DNA molecules as well as functional nucleic acids in general. If it is not, it still raises the intriguing possibility of finding the best sequence or global optimum with respect to a specific functional motif such as Aurora.

## Supplementary Material

gkaf1348_Supplemental_File

## Data Availability

Sequencing data has been deposited to BioStudies under accession number S-BSST2257 (https://www.ebi.ac.uk/biostudies/studies/S-BSST2257). A copy of the code has been deposited to Figshare at https://doi.org/10.6084/m9.figshare.30490904.
