# Supplementary material for: Using structured libraries, selection, and machine learning to rapidly explore the sequence space of a fluorescent deoxyribozyme
Source: Nucleic Acids Res. 2025 Dec 12;53(22):gkaf1348. doi: 10.1093/nar/gkaf1348 (PMC12700094; doi:10.1093/nar/gkaf1348)
Supplement: gkaf1348_Supplemental_File [file gkaf1348_supplemental_file.pdf]

Using structured libraries, selection and machine learning to rapidly explore the sequence space of a fluorescent deoxyribozyme

Jaroslav Kurfürst[†1,2], Martin Volek[†1,3], Raman Samusevich[1], Tomáš Pluskal[1], and Edward A. Curtis[1*]

[1]Institute of Organic Chemistry and Biochemistry of the Czech Academy of Sciences, Prague 166 10, Czech Republic

[2]Department of Informatics and Chemistry, University of Chemistry and Technology, Prague 166 28, Czech Republic

[3]Department of Genetics and Microbiology, Faculty of Science, Charles University in Prague, Prague 128 44, Czech Republic

SUPPLEMENTARY INFORMATION

Supplementary Table 1 | Sequences of deoxyribozymes and oligonucleotides used in this study.

**Name**	**Nucleotide sequence**
Aurora 2	GGAAGGGATGACTATGTCCGGTTCCTGTAAGGCATGTG GAGTGTTGT
Aurora 34G	GGAAGGGATGAATATGTCCGGTTCCAGTATGGCGTGTG GAGTGTTTT
Aurora 34T	GGAAGGGATGAATATGTCCGGTTCCAGTATGGCTTGTG GAGTGTTTT
FWD1	ACCGCTCAGGTGTAGTATCA
Splint1	GTCGCCATCTCTTCCTGATACTACACCTGAGCGGT
FWD1r	ACCGCTCAGGTGTAGTATCrA
REV1	CATCGGGCAGATCATTAGTG
REV1p	pCATCGGGCAGATCATTAGTG
Au_secondary structured_SubPool_1	GGAGGRGATGRATRYRYCCGGKHCCRKWWYGGCDTGH GGRGYRYATYAACACTAATGATCTGCCCGATG
Au_secondary structured_SubPool_2	GGAAGRGATGRATRYRYCCGGKHCCRKWWYGGCDTGH GGRGYRYTTYAACACTAATGATCTGCCCGATG
Au_secondary structured_SubPool_3	GGAGGRGATGRCTRYRYCCGGKHCCRKWWYGGCDTGH GGRGYRYAGYAACACTAATGATCTGCCCGATG
Au_secondary structured_SubPool_4	GGAGGRGATGRATRYRYCCGGKHCCYKWWRGGCDTGH GGRGYRYATYAACACTAATGATCTGCCCGATG
Au_secondary structured_SubPool_5	GGAAGRGATGRCTRYRYCCGGKHCCRKWWYGGCDTGH GGRGYRYTGYAACACTAATGATCTGCCCGATG
Au_secondary structured_SubPool_6	GGAAGRGATGRATRYRYCCGGKHCCYKWWRGGCDTGH GGRGYRYTTYAACACTAATGATCTGCCCGATG
Au_secondary structured_SubPool_7	GGAGGRGATGRCTRYRYCCGGKHCCYKWWRGGCDTGH GGRGYRYAGYAACACTAATGATCTGCCCGATG
Au_secondary structured_SubPool_8	GGAAGRGATGRCTRYRYCCGGKHCCYKWWRGGCDTGH GGRGYRYTGYAACACTAATGATCTGCCCGATG
Au_random_SubPool_1	GGAMGGGAKGABTMTGTCCKKTDCCTKKWMRGYVTRY RKAGTGKTGYAACACTAATGATCTGCCCGATG
Au_random_SubPool_2	GCAMGGGAKGGBTMTGTCCKKTDCCTKKWMRGYVTRY RKAGTGKTGYAACACTAATGATCTGCCCGATG
Au_random_SubPool_3	GGAMGGGAKGABTMTGTCAKKTDCCTKKWMRGYVTRYR KATTGKTGYAACACTAATGATCTGCCCGATG
Au_random_SubPool_4	GGAMGGGAKGABTMTGTCCKKTDCCTKYWMRGYVTRY RKAGTGSTGYAACACTAATGATCTGCCCGATG
Au_random_SubPool_5	GCAMGGGAKGGBTMTGTCAKKTDCCTKKWMRGYVTRY RKATTGKTGYAACACTAATGATCTGCCCGATG

Au_random_SubPool_6	GCAMGGGAKGGBTMTGTCCKKTDCCTKYWMRGYVTRYRKAGTGSTGYAACACTAATGATCTGCCCGATG
Au_random_SubPool_7	GGAMGGGAKGABTMTGTCAKKTDCCTKYWMRGYVTRYRKATTGSTGYAACACTAATGATCTGCCCGATG
Au_random_SubPool_8	GCAMGGGAKGGBTMTGTCAKKTDCCTKYWMRGYVTRYRKATTGSTGYAACACTAATGATCTGCCCGATG
Au_secondary_structured_20μM_selection_Hit1	GGAAGGGATGAATATGTCCGGTACCAGTATGGCGTGTGGAGTGTTTT
Au_secondary_structured_20μM_selection_Hit2	GGAAGGGATGAATATGTCCGGGTCCTGTAGGGCGTGCGGAGTGTTTT
Au_secondary_structured_20μM_selection_Hit3	GGAAGAGATGAATATGTCCGGGACCCTTTGGGCGTGTGGAGTGTTTT
Au_secondary_structured_20μM_selection_Hit4	GGAAGGGATGAATATGTCCGGTACCTTTAAGGCGTGAGGAGTGTTTT
Au_secondary_structured_20μM_selection_Hit5	GGAGGAGATGGATATGTCCGGTTCCTGAAAGGCGTGTGGAGTGTATT
Au_secondary_structured_20μM_selection_Hit6	GGAAGGGATGAATATGTCCGGTACCTGTAAGGCGTGCGGAGCGTTTT
Au_secondary_structured_20μM_selection_Hit7	GGAAGAGATGAATATGTCCGGTTCCAGTATGGCGTGAGGAGTGTTTC
Au_secondary_structured_20μM_selection_Hit8	GGAAGAGATGGATATGTCCGGGCCCCTTTGGGCGTGTGGAGCGTTTT
Au_secondary_structured_20μM_selection_Hit9	GGAAGGGATGGATATGCCCGGTTCCTGTAAGGCGTGTGGGGTGTTTC
Au_secondary_structured_20μM_selection_Hit10	GGAAGGGATGGATATGTCCGGGACCAGAATGGCGTGTGGAGTGTTTC
Au_secondary_structured_20μM_selection_Hit11	GGAGGAGATGGATATGTCCGGGACCCTATGGGCTTGTGGAGTGTATC
Au_secondary_structured_20μM_selection_Hit12	GGAGGAGATGGATATGTCCGGTACCTTTTAGGCGTGTGGAGCGTATT
Au_secondary_structured_20μM_selection_Hit13	GGAAGGGATGAATATGTCCGGTACCCGTAGGGCGTGCGGAGTGTTTT
Au_secondary_structured_20μM_selection_Hit14	GGAAGAGATGAATATGTCCGGTTCCTGAAAGGCGTGAGGAGTGTTTC
Au_secondary_structured_20μM_selection_Hit15	GGAAGGGATGGATATGTCCGGGACCCTTTGGGCGTGTGGAGTGTTTT
Au_secondary_structured_20μM_selection_Hit16	GGAAGGGATGGATATGTCCGGGTCCCGAAGGGCGTGTGGAGTGTTTC
Au_secondary_structured_20μM_selection_Hit17	GGAAGAGATGAATATGTCCGGTTCCGTTTCGGCGTGTGGAGTGTTTC
Au_secondary_structured_20μM_selection_Hit18	GGAAGAGATGAATATGTCCGGGTCCAGAATGGCTTGTGGAGTGTTTT

Au_secondary_structured_20μM_selection_Hit19	GGAAGAGATGAATATGTCCGGTTCCATTTTGGCATGTGGAGTGTTTT
Au_secondary_structured_20μM_selection_Hit20	GGAAGAGATGGATATGTCCGGTTCCGGTACGGCGTGTGGAGTGTTTC
Au_secondary_structured_20μM_selection_Hit21	GGAAGAGATGGATATGTCCGGGTCCCTTAGGGCGTGCGGAGCGTTTT
Au_secondary_structured_20μM_selection_Hit22	GGAAGGGATGAATATGTCCGGTTCCTTTAAGGCGTGAGGAGCGTTTC
Au_secondary_structured_20μM_selection_Hit23	GGAAGGGATGAATATGTCCGGTACCATTATGGCGTGAGGAGTGTTTC
Au_secondary_structured_20μM_selection_Hit24	GGAAGGGATGGATATGTCCGGTTCCATTTTGGCTTGTGGAGTGTTTC
Au_secondary_structured_20μM_selection_Hit25	GGAAGAGATGGATATGTCCGGTCCCAGTTTGGCTTGTGGAGTGTTTC
Au_secondary_structured_20μM_selection_Hit26	GGAAGAGATGAATATGCCCGGTACCATTATGGCTTGTGGGGTGTTTT
Au_secondary_structured_20μM_selection_Hit27	GGAAGGGATGGATATGTCCGGGTCCAGATTGGCGTGTGGAGCGTTTT
Au_secondary_structured_20μM_selection_Hit28	GGAAGAGATGAATGTATCCGGTACCGTTTTGGCATGCGGGGTGTTTT
Au_secondary_structured_20μM_selection_Hit29	GGAAGAGATGAATACACCCGGGACCATTTTGGCATGTGGAGTGTTTC
Au_secondary_structured_20μM_selection_Hit30	GGAGGAGATGAATACATCCGGTACCAGTTCGGCTTGAGGAGTATATC
Au_secondary_structured_100μM_selection_Hit1	GGAAGGGATGAATATGTCCGGTTCCAGTATGGCTTGTGGAGTGTTTT
Au_secondary_structured_100μM_selection_Hit2	GGAAGGGATGAATATGTCCGGTACCGTTTTGGCTTGTGGAGCGTTTT
Au_secondary_structured_100μM_selection_Hit3	GGAAGGGATGAATATGTCCGGGCCCCTATGGGCATGTGGAGCGTTTT
Au_secondary_structured_100μM_selection_Hit4	GGAAGGGATGAATATGTCCGGTCCCATTATGGCGTGTGGAGTGTTTT
Au_secondary_structured_100μM_selection_Hit5	GGAAGGGATGAATATGTCCGGGTCCTTTTAGGCGTGTGGAGCGTTTC
Au_secondary_structured_100μM_selection_Hit6	GGAAGGGATGAATATGTCCGGGCCCCTTTGGGCTTGCGGAGCGTTTT
Au_secondary_structured_100μM_selection_Hit7	GGAAGAGATGGATATGTCCGGTCCCATTATGGCGTGTGGAGTGTTTT
Au_secondary_structured_100μM_selection_Hit8	GGAAGGGATGAATATGTCCGGGTCCCTTAGGGCGTGAGGAGCGTTTT
Au_secondary_structured_100μM_selection_Hit9	GGAAGGGATGAATATGTCCGGTACCTTATAGGCGTGAGGAGCGTTTC

Au_secondary_structured_100μM_selection_Hit10	GGAAGGGATGAATATGCCCGGTTCCCTTTAGGCATGCGGGGTGTTTT
Au_secondary_structured_100μM_selection_Hit11	GGAAGAGATGAATATGTCCGGTTCCCGTAGGGCTTGTGGAGCGTTTT
Au_secondary_structured_100μM_selection_Hit12	GGAAGAGATGAATATGCCCGGTACCAGAATGGCATGAGGGGTGTTTT
Au_secondary_structured_100μM_selection_Hit13	GGAAGGGATGAATATGTCCGGTTCCATTTTGGCGTGCGGAGCGTTTT
Au_secondary_structured_100μM_selection_Hit14	GGAAGAGATGGATATGTCCGGTCCCTGTAAGGCATGTGGAGTGTTTC
Au_secondary_structured_100μM_selection_Hit15	GGAAGGGATGGATATGCCCGGTACCAGTTTGGCGTGTGGGGTGTTTT
Au_secondary_structured_100μM_selection_Hit16	GGAAGGGATGAATATGCCCGGTACCGGTACGGCTTGTGGGGCGTTTT
Au_secondary_structured_100μM_selection_Hit17	GGAAGAGATGGATATGTCCGGTACCTTTAAGGCGTGCGGAGTGTTTC
Au_secondary_structured_100μM_selection_Hit18	GGAAGAGATGAATATGTCCGGTCCCTTTTAGGCATGCGGAGCGTTTT
Au_secondary_structured_100μM_selection_Hit19	GGAAGAGATGAATATGCCCGGTCCCTTTTAGGCATGCGGGGTGTTTC
Au_secondary_structured_100μM_selection_Hit20	GGAAGAGATGGATATGCCCGGTTCCAGTTTGGCTTGAGGGGTGTTTT
Au_secondary_structured_100μM_selection_Hit21	GGAGGAGATGGATATGCCCGGTTCCAGAATGGCATGTGGGGTGTATC
Au_secondary_structured_100μM_selection_Hit22	GGAAGAGATGAATATGCCCGGGTCCGGAATGGCGTGTGGGGTGTTTT
Au_secondary_structured_100μM_selection_Hit23	GGAGGAGATGGATATGTCCGGTTCCAGTATGGCATGCGGAGTGTATC
Au_secondary_structured_100μM_selection_Hit24	GGAAGAGATGAATATGTCCGGGACCCGTAGGGCGTGTGGAGCGTTTC
Au_secondary_structured_100μM_selection_Hit25	GGAAGAGATGGATATGTCCGGTACCAGTTTGGCATGCGGAGTGTTTT
Au_secondary_structured_100μM_selection_Hit26	GGAAGAGATGAATATGCCCGGGACCATTATGGCGTGTGGGGTGTTTC
Au_secondary_structured_100μM_selection_Hit27	GGAAGAGATGAATATGCCCGGGACCAGTTTGGCATGTGGGGTGTTTC
Au_secondary_structured_100μM_selection_Hit28	GGAAGAGATGAATATGTCCGGGACCGGTTTGGCGTGAGGAGTGTTTC
Au_secondary_structured_100μM_selection_Hit29	GGAAGGGATGAATGTACCCGGGTCCCTTAGGGCATGAGGAGTGTTTT
Au_secondary_structured_100μM_selection_Hit30	GGAGGAGATGAATGTGTCCGGTACCGTATCGGCATGTGGGGCATATT

Au_random_20uM_selection_Hit1	GGAAGGGATGACTATGTCCGGTTCCTTTTAGGCGTGTGGAGTGTTGT
Au_random_20μM_selection_Hit2	GGAAGGGATGACTATGTCCGGTTCCTGTAAGGCGTGTGGAGTGTTGT
Au_random_20μM_selection_Hit3	GGAAGGGATGACTATGTCCGGTTCCTGTTAGGCGTGTGGAGTGTTGT
Au_random_20μM_selection_Hit4	GGAAGGGATGACTATGTCCGGTTCCTTTAAGGCGTGTGGAGTGTTGT
Au_random_20μM_selection_Hit5	GGAAGGGATGACTATGTCCGGTACCTTTTAGGCGTGTGGAGTGTTGT
Au_random_20μM_selection_Hit6	GGAAGGGATGACTATGTCCGGTACCTGTAAGGCGTGTGGAGTGTTGT
Au_random_20μM_selection_Hit7	GGAAGGGATGACTATGTCCGGTTCCTGTAAGGCGTGCGGAGTGTTGT
Au_random_20μM_selection_Hit8	GGAAGGGATGACTATGTCCGGTTCCTTTTAGGCGTGCGGAGTGTTGT
Au_random_20μM_selection_Hit9	GGAAGGGATGACTATGTCCGGTTCCTGTTAGGCGTGCGGAGTGTTGT
Au_random_20μM_selection_Hit10	GGAAGGGATGACTATGTCCGGTACCTTTAAGGCGTGTGGAGTGTTGT
Au_random_20μM_selection_Hit11	GGAAGGGATGACTATGTCCGGTACCTGTTAGGCGTGTGGAGTGTTGT
Au_random_20μM_selection_Hit12	GGAAGGGATGACTATGTCCGGTTCCTGTAAGGCATGTGGAGTGTTGT
Au_random_20μM_selection_Hit13	GGAAGGGATGACTATGTCCGGTTCCTTTAAGGCGTGCGGAGTGTTGT
Au_random_20μM_selection_Hit14	GGAAGGGATGACTATGTCCGGTTCCTTTTAGGCATGTGGAGTGTTGT
Au_random_20μM_selection_Hit15	GGAAGGGATGACTATGTCCGGTTCCTTTTAGGCGTGTGGAGTGTTGC
Au_random_20μM_selection_Hit16	GGAAGGGATGACTATGTCCGGTTCCTGTAAGGCGTGTGGAGTGTTGC
Au_random_20μM_selection_Hit17	GGAAGGGATGACTATGTCCGGTACCTTTTAGGCGTGCGGAGTGTTGT
Au_random_20μM_selection_Hit18	GGAAGGGATGACTATGTCCGGTTCCTGTTAGGCGTGTGGAGTGTTGC
Au_random_20μM_selection_Hit19	GGAAGGGATGACTATGTCCGGTTCCTGTTAGGCATGTGGAGTGTTGT

Supplementary Table 2 | Numbers of enriched sequences in the structured vs. control libraries, for cases where all sequences are considered, or when sequences common to the structured and random libraries are filtered out.

	All sequences		Common sequences filtered	
**CPM threshold**	**structured**	**random**	**structured**	**random**
**0.1**	1910646	2039565	1910565	2039489
**1.0**	50539	141951	50478	141897
**10.0**	6585	117	6538	80
**100.0**	245	12	229	0

Supplementary Table 3 | Detailed description of random splits and sampling sizes.

sampling	train_size	test_size
0.3	2251137	2251137
0.1	750379	750379
0.03	225113	225113
0.01	75037	75037
0.003	22511	22511
0.001	7503	7503

Supplementary Table 4 | Detailed description of explore splits and sampling sizes.

sampling	test_size	val_size	train_size
1	100	100	7503593
0.1	100	100	750359
0.01	100	100	75036
0.001	100	100	7504

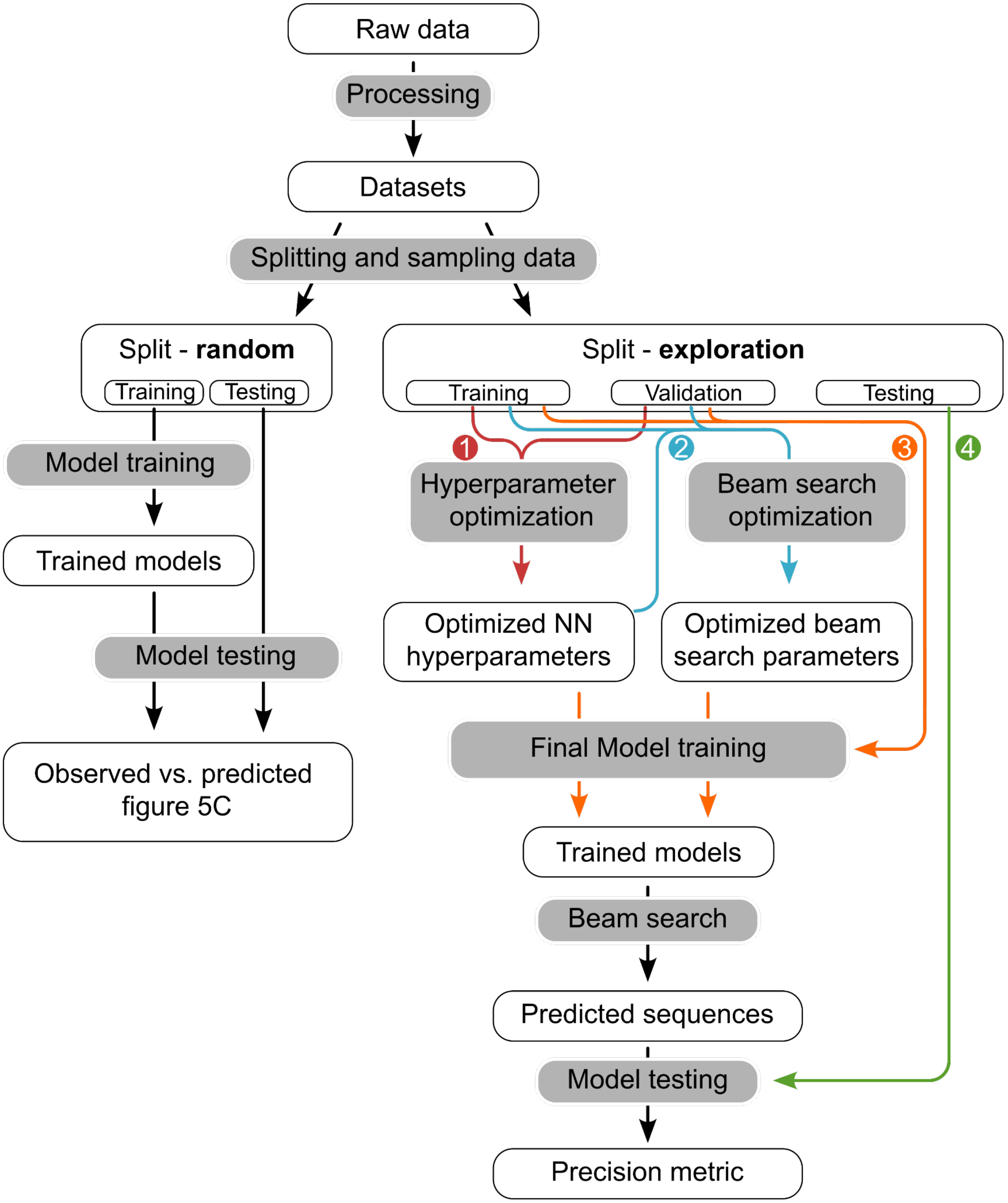

Supplementary Fig. 1 | Data-flow scheme. See the explanation in the supplementary methods for more information.

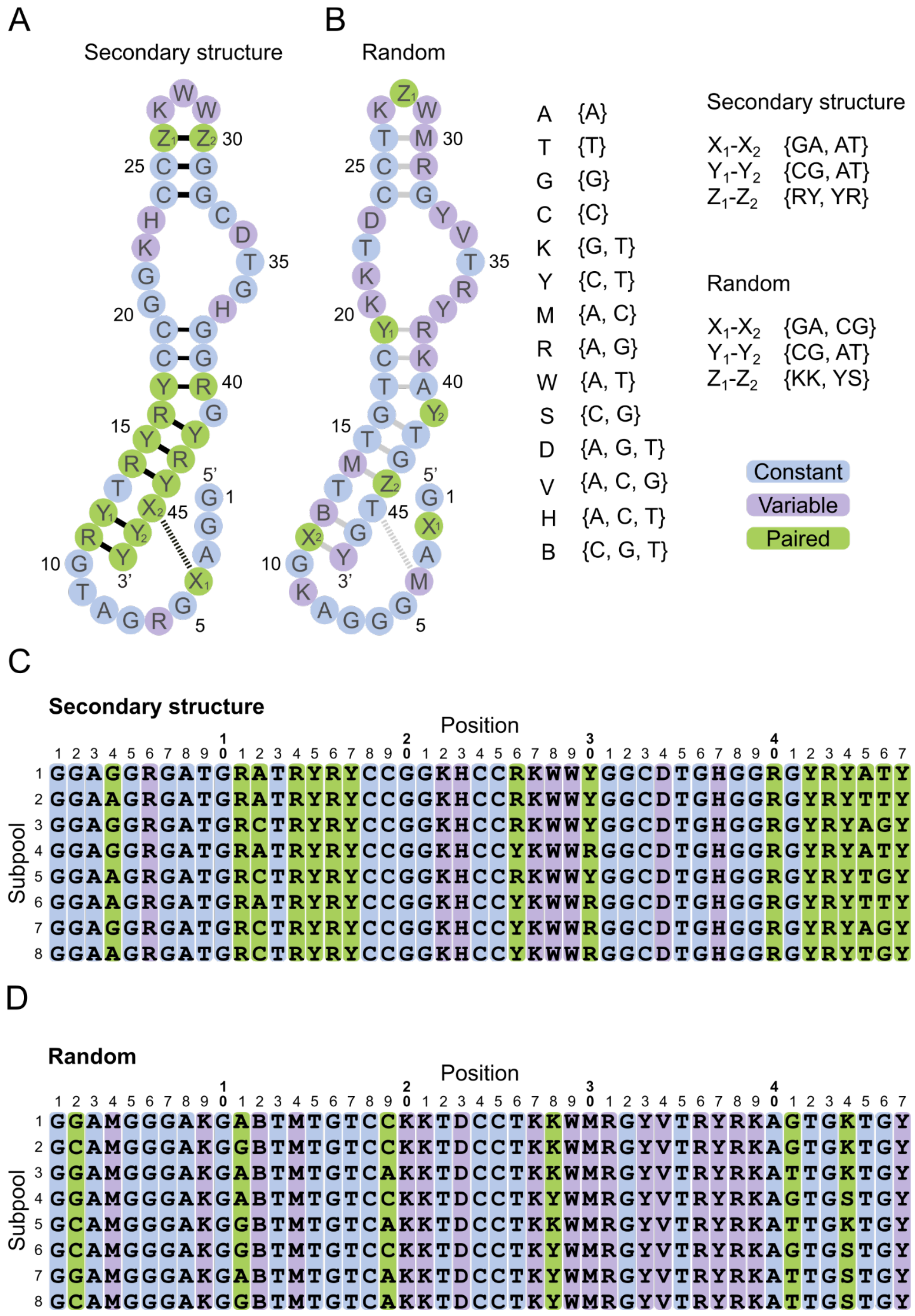

Supplementary Fig. 2 | Structured and randomly mutagenized libraries. A) Design of the secondary structure library. B) Design of the randomly mutagenized library. C) Sequences of the eight degenerate oligonucleotides used to construct the secondary structure library. D) Sequences of the eight degenerate oligonucleotides used to construct the randomly mutagenized library.

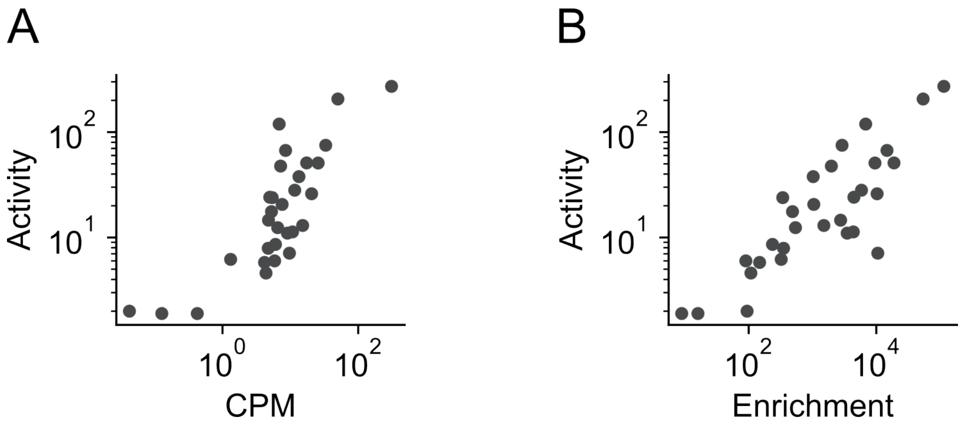

Supplementary Fig. 3 | Ranking sequences using CPM and enrichment values. A) Relationship between CPM value and catalytic activity of sequences from the secondary structure library after selection. B) Relationship between enrichment value and catalytic activity of sequences from the secondary structure library after selection. Activities are expressed relative to Aurora 2.

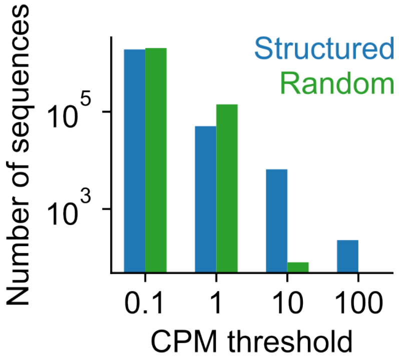

Supplementary Fig. 4 | Number of sequences above different CPM thresholds in the structured and control libraries. Unlike the dataset used to make Figure 2g, these datasets do not contain the 128 sequences encoded by both libraries.

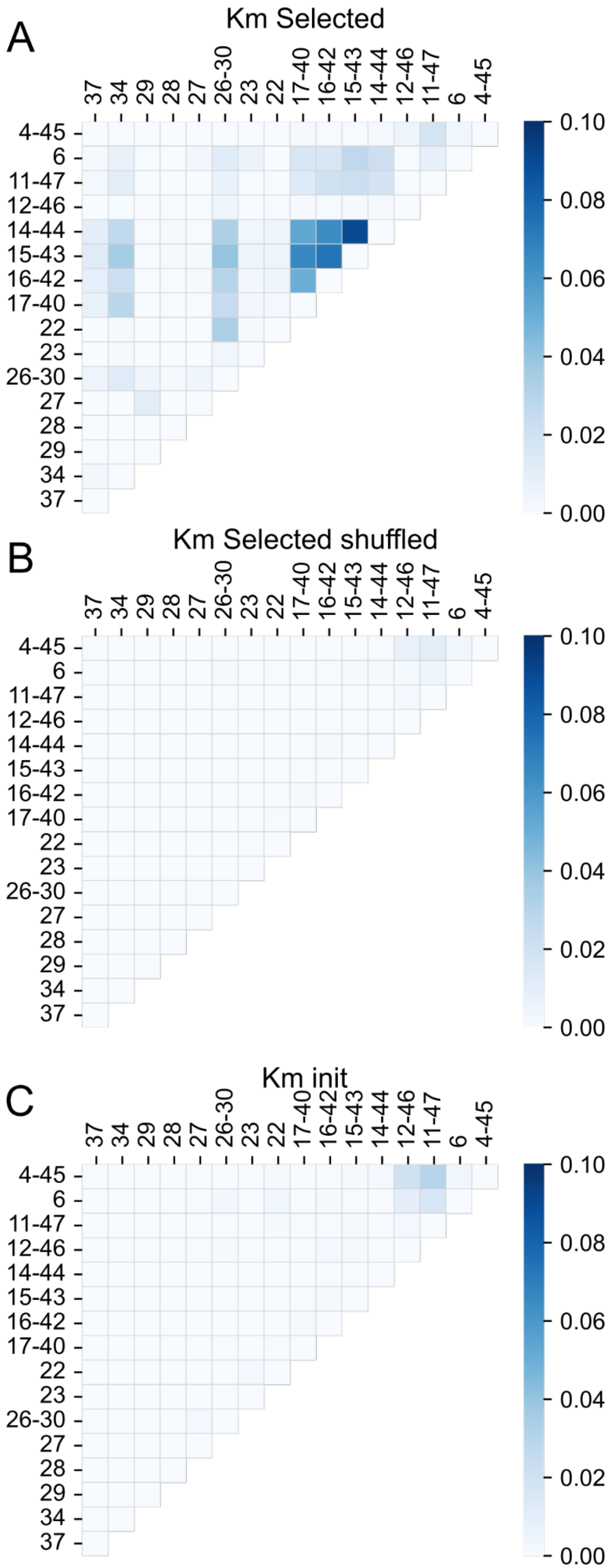
A
Km Selected
37
34
29
28
27
26-30
23
22
17-40
16-42
15-43
14-44
12-46
11-47
6
4-45
0.10
0.08
0.06
0.04
0.02
0.00
B
Km Selected shuffled
C
Km init

Supplementary Fig. 5 | Mutual information analysis. A) Mutual information between pairs of positions in the secondary structure library after selection for improved K_M values. B) Mutual information between pairs of positions in a control dataset in which sequence and CPM values in the selected library were randomly shuffled. C) Mutual information between pairs of positions in the starting library. In each case, base pairs and the 4-45 noncanonical pair were treated as units.

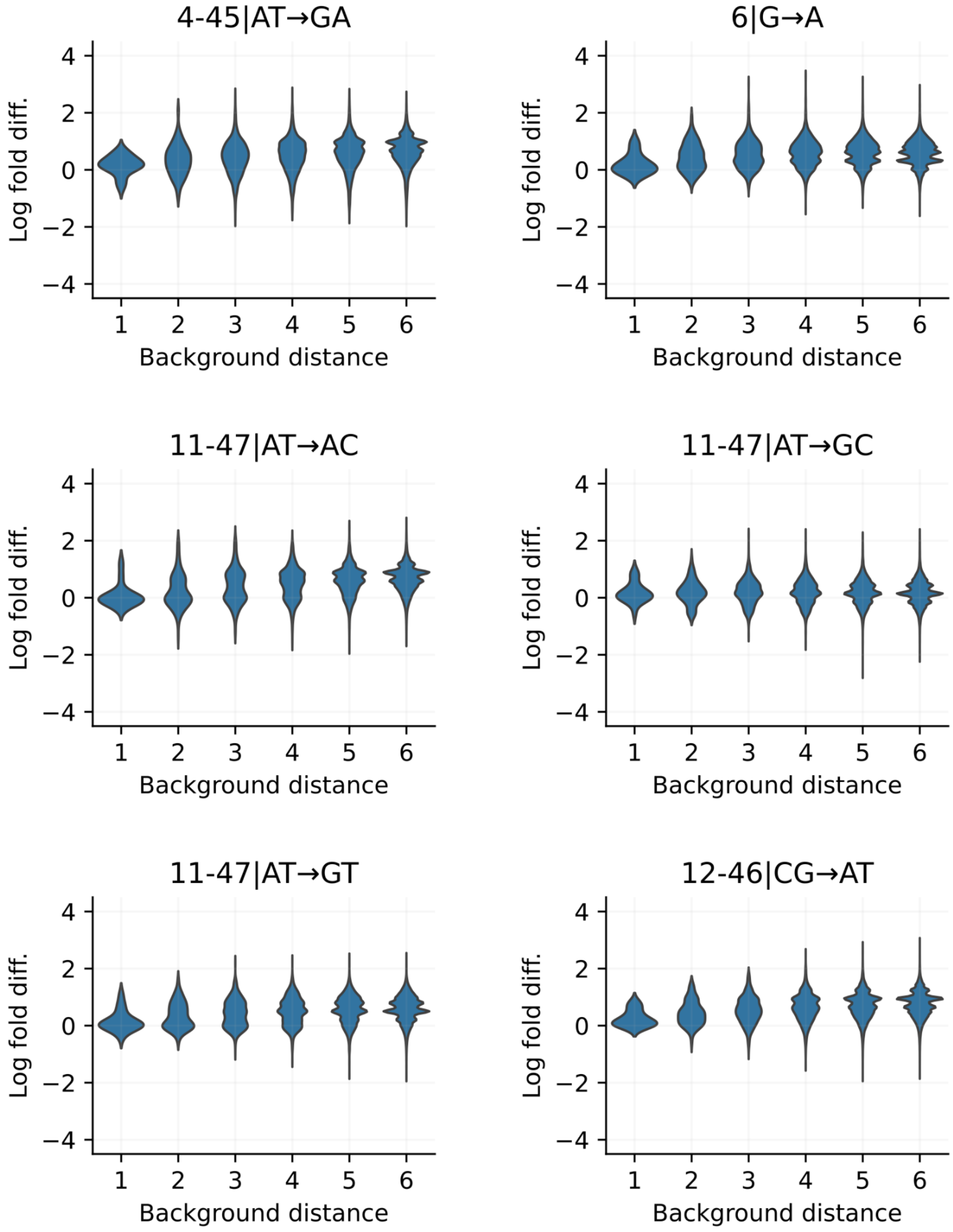
4-45|AT→GA
6|G→A
11-47|AT→AC
11-47|AT→GC
11-47|AT→GT
12-46|CG→AT
Log fold diff.
Background distance
4
2
0
−2
−4
1
2
3
4
5
6

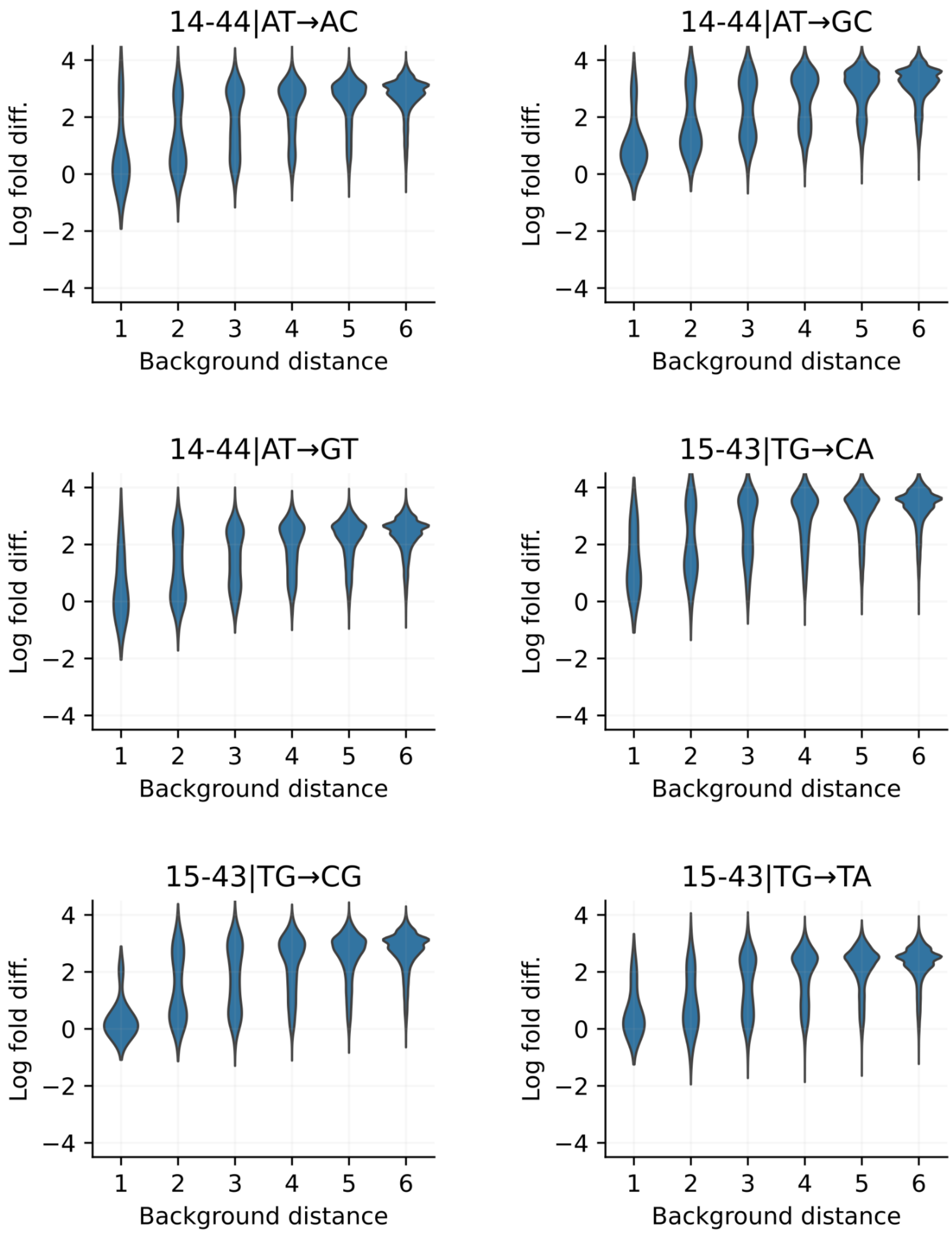
14-44|AT→AC
14-44|AT→GC
14-44|AT→GT
15-43|TG→CA
15-43|TG→CG
15-43|TG→TA
Log fold diff.
Background distance
−4
−2
0
2
4
1
2
3
4
5
6

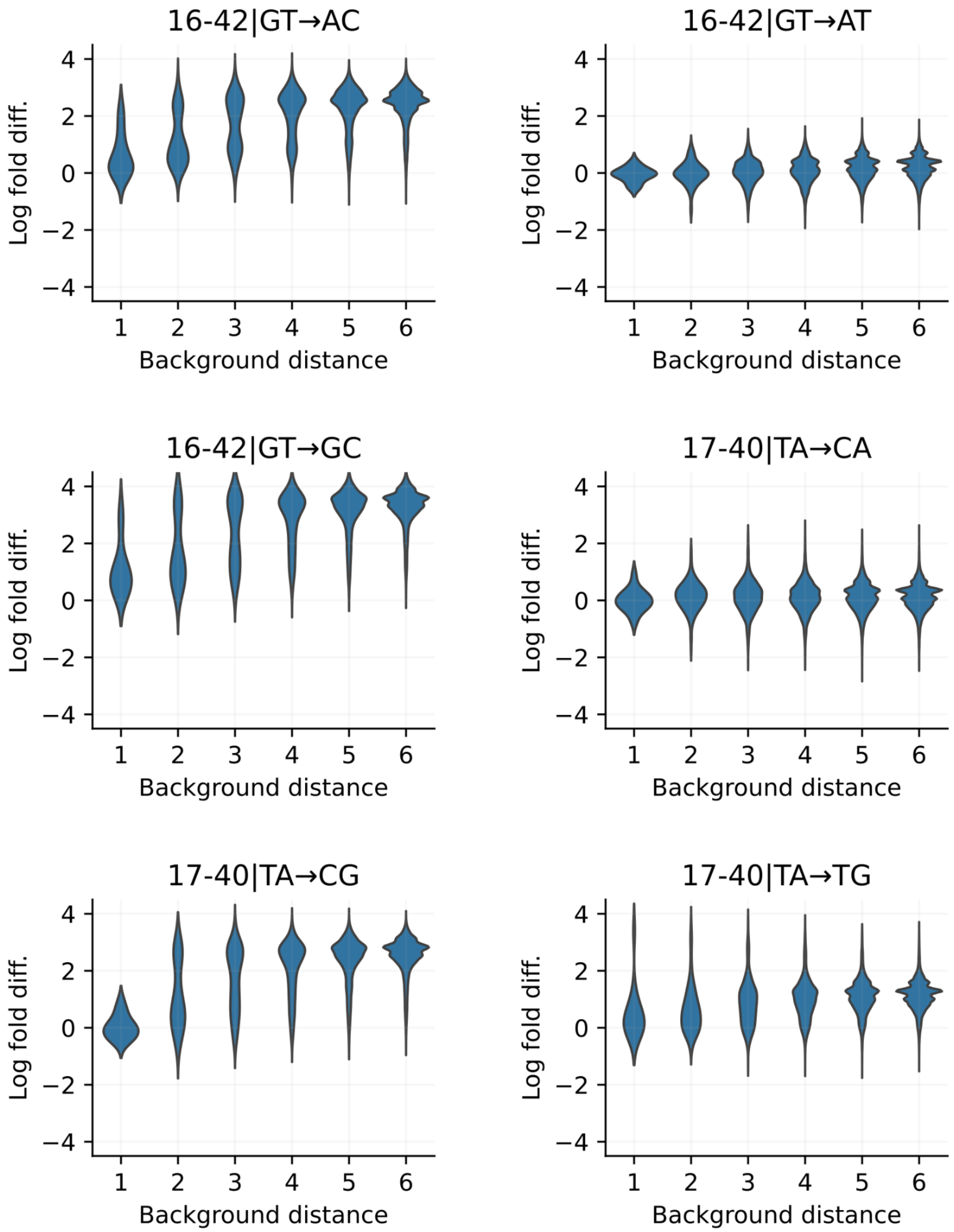

16-42|GT→AC
16-42|GT→AT
16-42|GT→GC
17-40|TA→CA
17-40|TA→CG
17-40|TA→TG
Log fold diff.
Background distance

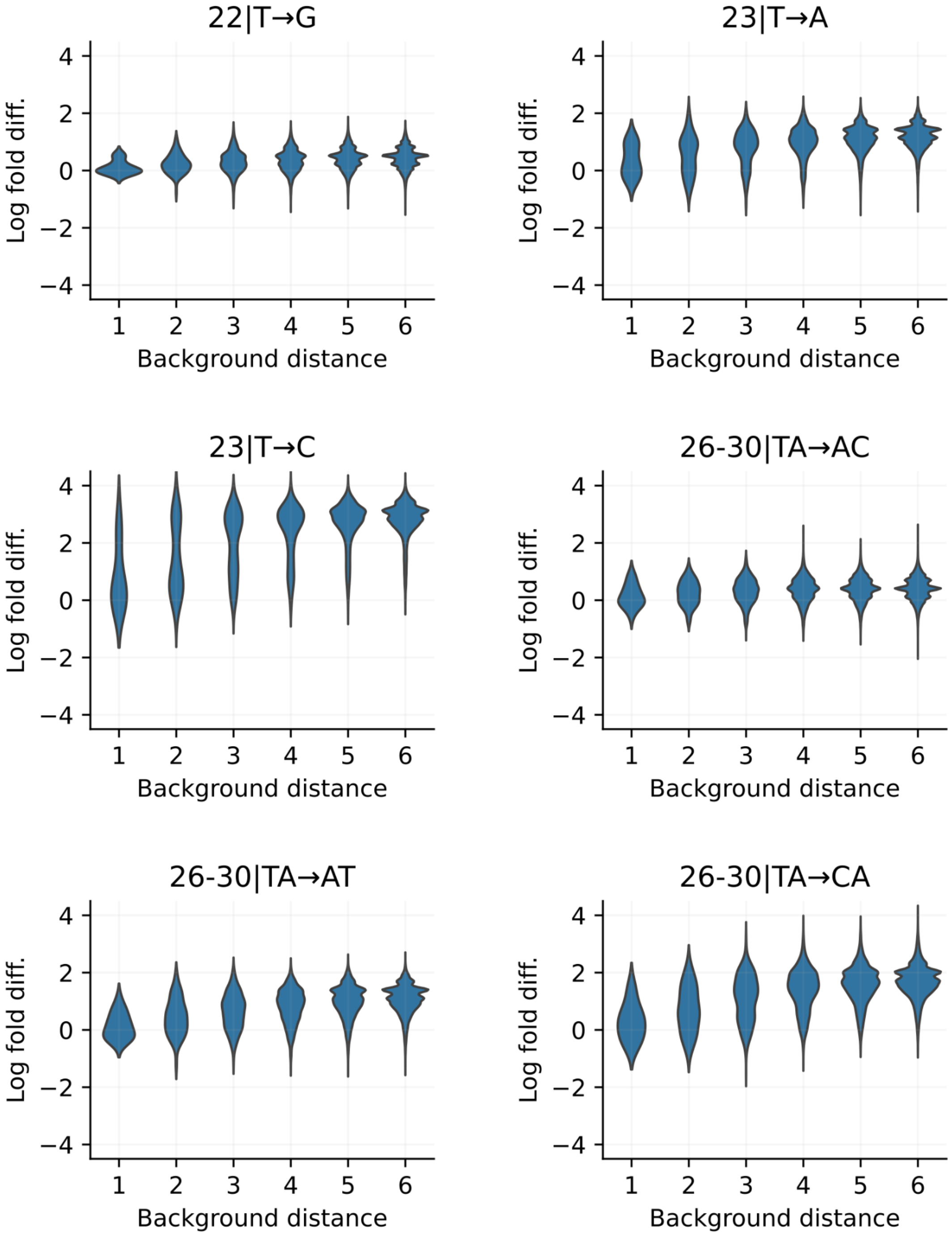
22|T→G
23|T→A
23|T→C
26-30|TA→AC
26-30|TA→AT
26-30|TA→CA
Log fold diff.
Background distance
−4
−2
0
2
4
1
2
3
4
5
6

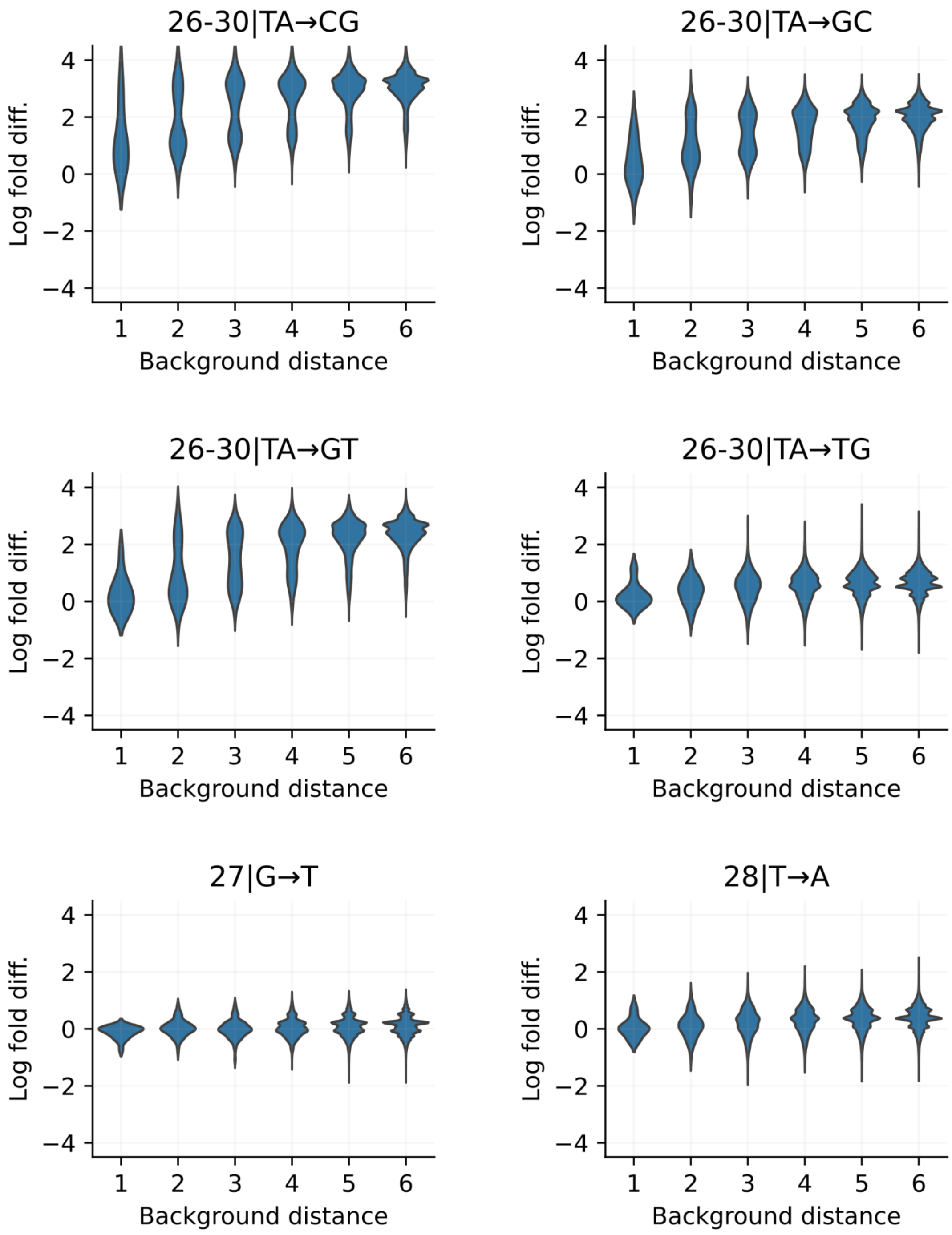
26-30|TA→CG
26-30|TA→GC
26-30|TA→GT
26-30|TA→TG
27|G→T
28|T→A
Log fold diff.
Background distance
4
2
0
−2
−4
1
2
3
4
5
6

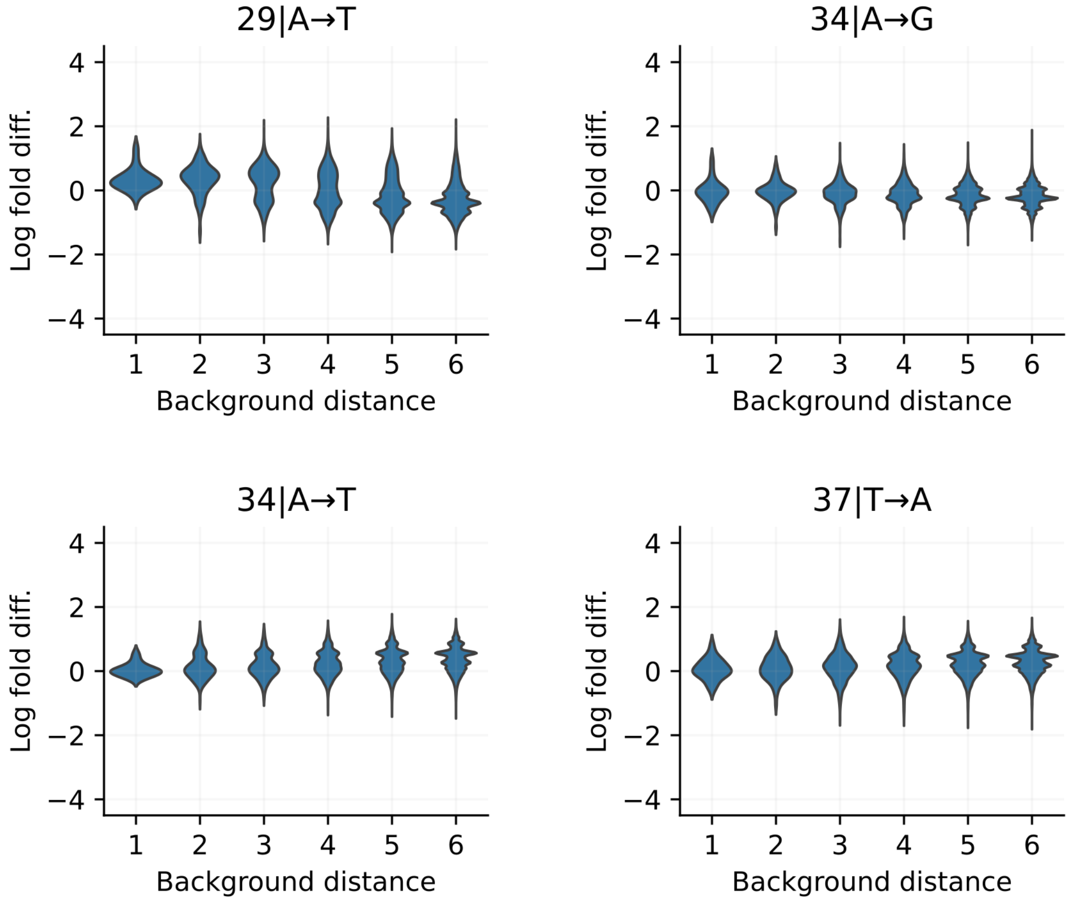

Supplementary Fig. 6 | Effect of mutational distance on the ability to predict mutational effects. Each plot shows analysis of a specific mutation at a single position (such as 6G to 6A) or base pair (such as 11-47 A-T to G-C). The x-axis shows the mutational distance of backgrounds from one another, and the y-axis shows the effect of the mutation relative to its effect in a reference mutational background (in this case Aurora 2). These plots indicate that the effects of mutations are typically easier to predict in backgrounds that are more similar to a reference sequence than in backgrounds that are less similar.

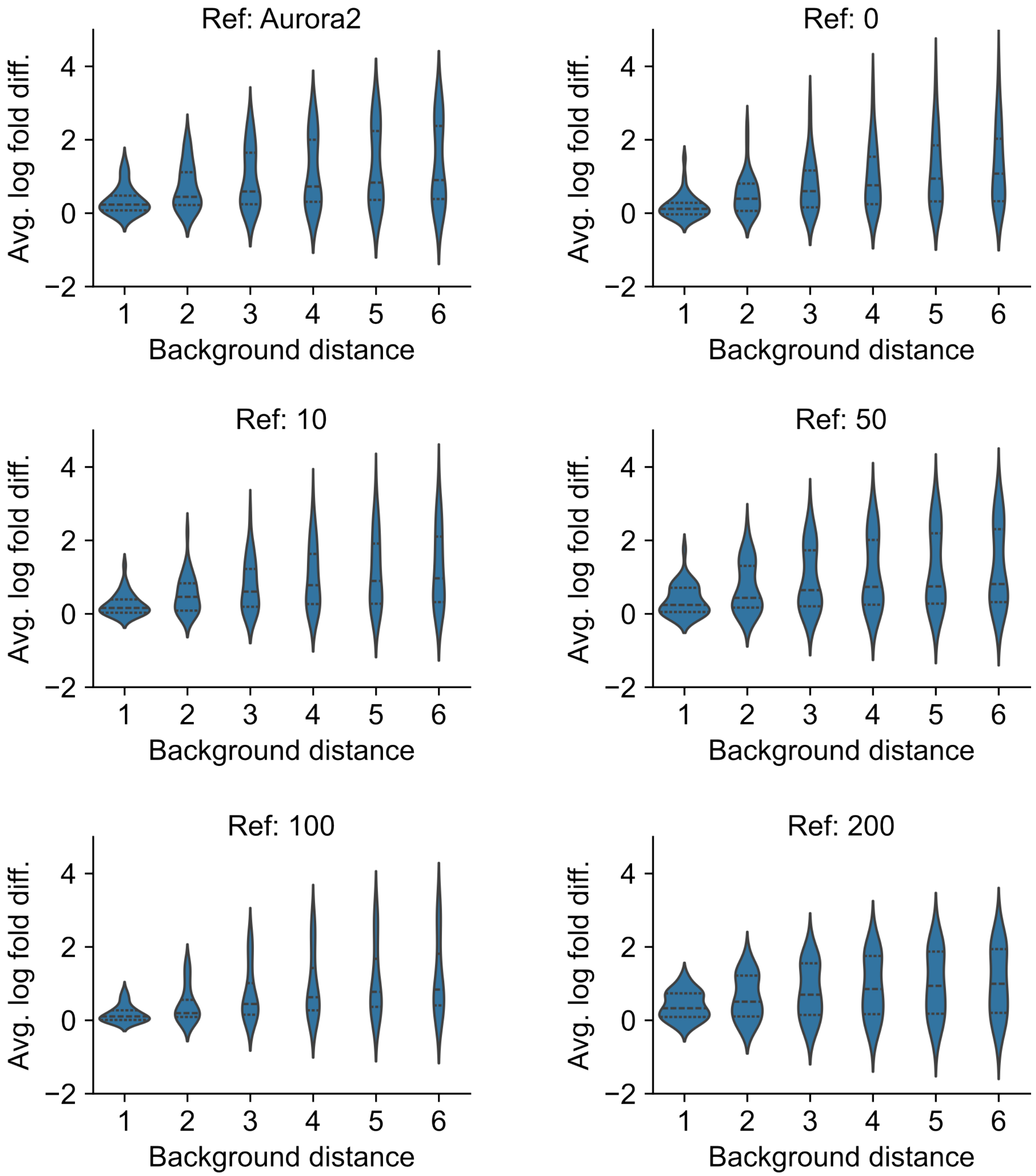

Supplementary Fig. 7 | Prediction of mutational effects in different backgrounds. Each plot shows the effect of every mutation encoded by the library in different mutational backgrounds relative to its effect in a reference background (such as Aurora 2). Backgrounds are sorted by their mutational distance from the reference background.

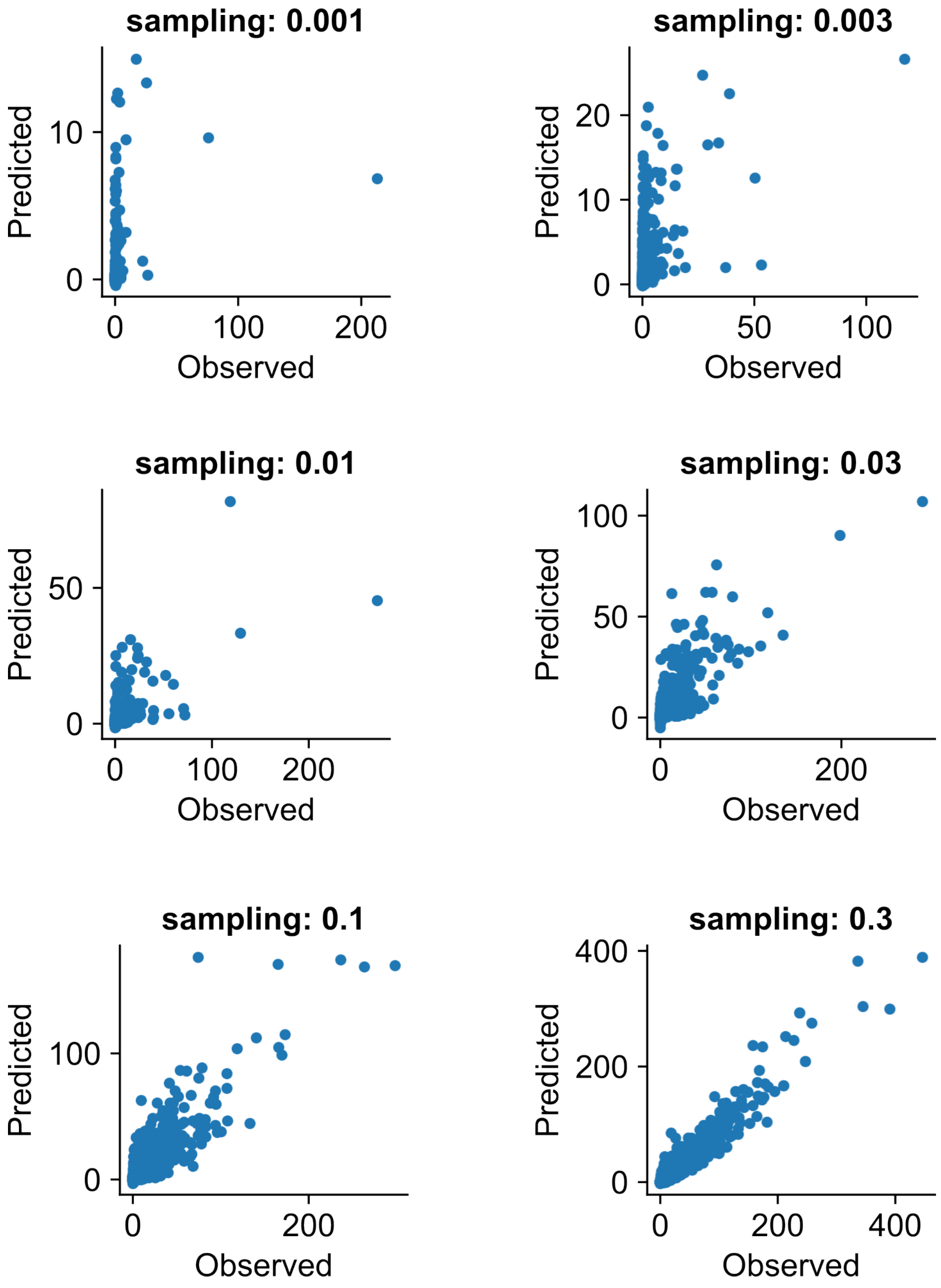

Supplementary Fig. 8 | Effect of sampling on the ability to model a fitness landscape. Each plot shows the correlation between observed and predicted CPM values for a training set of a different size. For example, "sampling: 0.001" indicates that 0.1% of the sequences in the dataset were used for the training set.

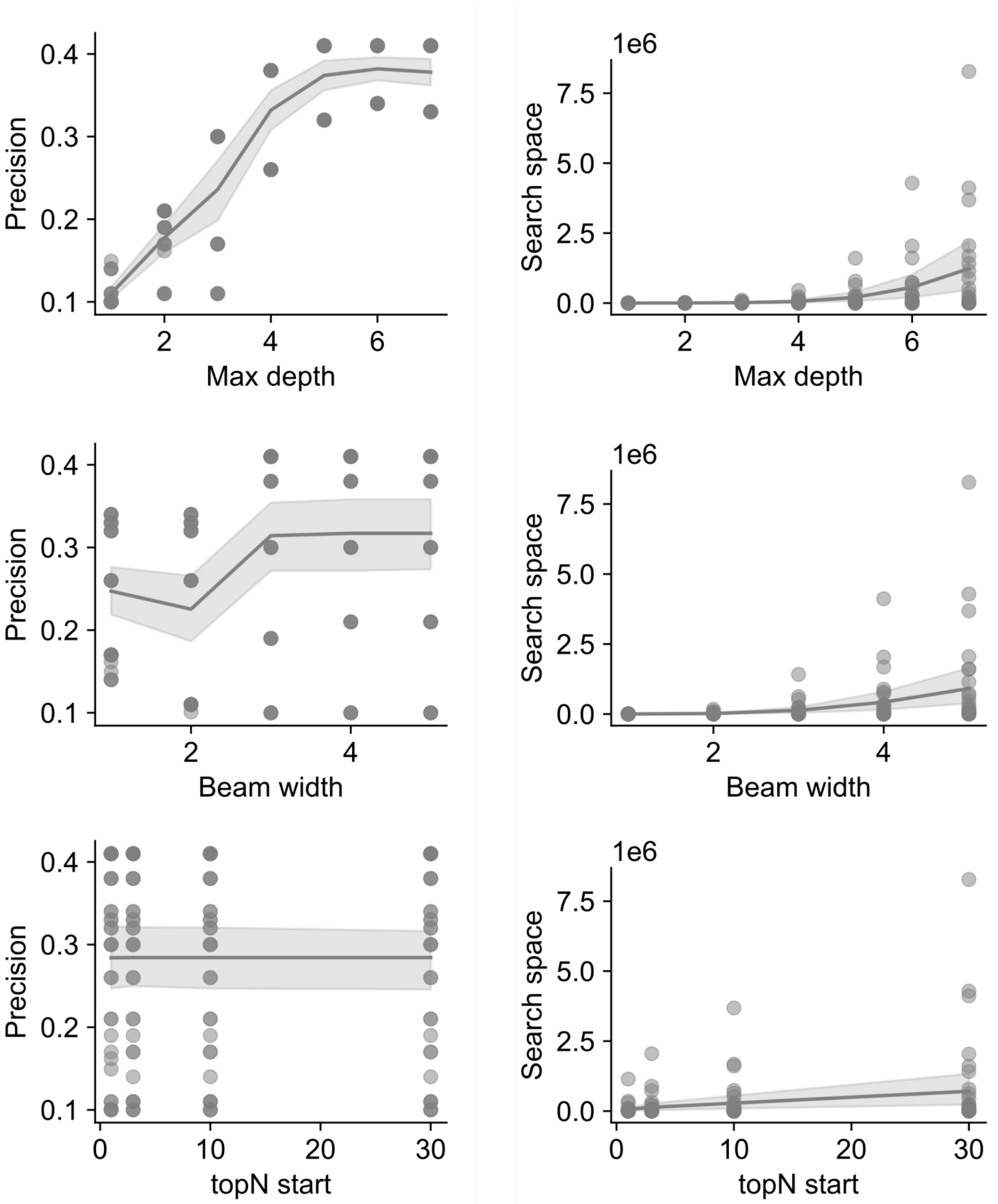

Supplementary Fig. 9 | Beam search parameter optimization. The top row shows the effect of the max_depth parameter of the beam search on precision (left) and the search space explored (right). The middle row shows the same, but for the beam_width parameter. The bottom row shows the same but for the topN_start parameter. These results show that max_depth is the only one of these three parameters that has a significant effect for this particular setup. They also indicate that reasonable precision can be obtained when only a fairly small space is explored. This can be achieved using a single start sequence, a beam_width value of 3, and a max_depth value between 5 and 6.

Supplementary Methods | A detailed description of the data-flow diagram shown in Supplementary Figure 1.

Raw data: paired-end, fastq files

Processing: Consists of: Adaptor trimming, read merging, read orientation unification, primer clipping, length filtering and counting of unique sequences.

Datasets: csv files with the following fields: “count” (Number of times a given unique sequence occurred in the data), “seq” (A full length unique sequence), “subpool”, “varseq” (Only variable positions extracted from the full sequence), “cpm” (Computed as count divided by the sum of all counts, then multiplied by a million).

Splitting and sampling data (random): Two different splits were made. The random split (left) consists of choosing a fraction of sequences randomly, once for training and once again for testing. This was done in such a way that the training and testing sets have no overlaps. Fractions of the following sizes (0.3, 0.1, 0.03, 0.01, 0.003, 0.001) were sampled from each group to examine how the sequence space sampling density affects the predictive capability of the model. Figure 5C was made with a model trained on the 0.1 fraction, the rest are shown in Supplementary Figure 6.

Splitting and sampling data (explore): The second split (right in Supplementary Figure 8) was made by taking the top 100 sequences as a testing set, and then taking the following top 100 sequences as a validation set. The rest of the sequences were used as the training set. This training set was then sampled in fractions of 1.0, 0.1, 0.01, or 0.001.

Model training (random): A fully connected neural network (MLPRegressor from the sklearn library with random_state=1, hidden_layer_sizes=(100, 100, 100, 100), activation='relu', batch_size=100, learning_rate_init=0.0001, early_stopping=True, validation_fraction=0.1, max_iter=40, learning_rate='constant, verbose=True) was used as the model to train on various samplings of the training data.

Model testing (random): The individual trained models were then used to predict CPM values of the testing sequences from corresponding samplings.

Hyperparameter optimization (exploration): A grid of the sklearn MLPRegressor hyperparameters was assembled. The values included in the grid search were: {'hidden_layer_sizes' : [(50, 50), (50, 50, 50), (100, 100), (100, 100, 100), (200, 200, 200), (100, 100, 100, 100)], 'learning_rate_init' : [0.01, 0.001, 0.0001], 'batch_size' : [100, 200]}. For each combination of values, a model was trained, beam search was then run with settings: 'topN_start' : 5, 'beam_width' : 5, 'max_depth' : 5, 'top_explored' : 100, 'mode' : 'directed'. Precision was

calculated with respect to the validation set. The best hyperparameter combination was then selected and used for the rest of the project.

Beam search optimization: With the NN hyperparameters optimized, we then investigated how the beam search parameters affect precision. The following values were used to construct a grid of all combinations: {'topN_start' : [5, 10, 20], 'beam_width' : [3, 6, 9], 'max_depth' : [6, 8, 10], 'top_explored': [100]}. This time, trained models were used to guide the beam search runs with various combinations of parameter values and precision was calculated with respect to the validation set. A single best combination of beam search parameters was selected for further use. Note, that this in particular is dataset-specific and will have to be optimized for every dataset. It might even be the case that 'optimizing' these parameters is not necessary and results instead primarily depend on the library design and selection outcomes.

Final model training: Once the optimized NN hyperparameters and optimized beam search parameters were determined, training and validation data were merged together to create a new training set. This training data was then used to train new models. These models were then used to guide the beam search with a single combination of parameters and precision was calculated with the predicted sequences and the testing set.
